# SALL2 represses cyclins D1 and E1 expression and restrains G1/S cell cycle transition and cancer‐related phenotypes

**DOI:** 10.1002/1878-0261.12308

**Published:** 2018-05-21

**Authors:** Viviana E. Hermosilla, Ginessa Salgado, Elizabeth Riffo, David Escobar, Matías I. Hepp, Carlos Farkas, Mario Galindo, Violeta Morín, María A. García‐Robles, Ariel F. Castro, Roxana Pincheira

**Affiliations:** ^1^ Departamento de Bioquímica y Biología Molecular Facultad de Ciencias Biológicas Universidad de Concepción Chile; ^2^ Millennium Institute on Immunology and Immunotherapy University of Chile Santiago Chile; ^3^ Program of Cellular and Molecular Biology Institute of Biomedical Sciences Faculty of Medicine University of Chile Santiago Chile; ^4^ Departamento de Biología Celular Facultad de Ciencias Biológicas Universidad de Concepción Chile

**Keywords:** cell cycle, cell proliferation, cyclin D1, cyclin E1, SALL2, tumorigenesis

## Abstract

SALL2 is a poorly characterized transcription factor that belongs to the Spalt‐like family involved in development. Mutations on *SALL2* have been associated with ocular coloboma and cancer. In cancers, SALL2 is deregulated and is proposed as a tumor suppressor in ovarian cancer. SALL2 has been implicated in stemness, cell death, proliferation, and quiescence. However, mechanisms underlying roles of SALL2 related to cancer remain largely unknown. Here, we investigated the role of SALL2 in cell proliferation using mouse embryo fibroblasts (MEFs) derived from *Sall2* ^*−/−*^ mice. Compared to *Sall2* ^*+*/*+*^ MEFs, *Sall2* ^*−*/*−*^ MEFs exhibit enhanced cell proliferation and faster postmitotic progression through G1 and S phases. Accordingly, *Sall2* ^*−/−*^ MEFs exhibit higher mRNA and protein levels of cyclins D1 and E1. Chromatin immunoprecipitation and promoter reporter assays showed that SALL2 binds and represses *CCND1* and *CCNE1* promoters, identifying a novel mechanism by which SALL2 may control cell cycle. In addition, the analysis of tissues from *Sall2* ^*+/+*^ and *Sall2* ^*−*/*−*^ mice confirmed the inverse correlation between expression of SALL2 and G1‐S cyclins. Consistent with an antiproliferative function of SALL2, immortalized *Sall2* ^*−*/*−*^ MEFs showed enhanced growth rate, foci formation, and anchorage‐independent growth, confirming tumor suppressor properties for SALL2. Finally, cancer data analyses show negative correlations between *SALL2* and G1‐S cyclins’ mRNA levels in several cancers. Altogether, our results demonstrated that SALL2 is a negative regulator of cell proliferation, an effect mediated in part by repression of G1‐S cyclins’ expression. Our results have implications for the understanding and significance of SALL2 role under physiological and pathological conditions.

**Abbreviations**
AP1: activator protein 1
ATF3: activating transcription factor 3
BrdU: 5‐bromo‐2′‐deoxyuridine
CCND1: cyclin D1 gene
CCNE1: cyclin E1 gene
CDK2: cyclin‐dependent kinase 2
CDK4/6: cyclin‐dependent kinase 4/6
CDK: cyclin‐dependent kinase
CDKN1A: cyclin‐dependent kinase inhibitor 1A gene
CDKN2A: cyclin‐dependent kinase inhibitor 2A gene
ChIP: chromatin immunoprecipitation
c‐MYC: v‐Myc avian myelocytomatosis viral oncogene homolog
E1A: exon 1A
E1: exon 1
G1/S: Gap1/synthesis
G1: Gap1
G2‐M: Gap2‐mitosis
HDAC: histone deacetylase
HOSE: human ovarian epithelial cells
HPV16 E6: human papillomavirus type 16 E6
iMEFs: immortalized mouse embryonic fibroblasts
LOH: loss of heterozygocity
MEFs: mouse embryonic fibroblasts
NFAT: nuclear factor of activated T cells
p16INK4a: cyclin‐dependent kinase inhibitor 2A
P1: promoter 1
p21WAF/CIP: cyclin‐dependent kinase inhibitor 1 or CDK interacting protein 1
P2: promoter 2
p53: p53 tumor suppressor protein
Ppib: peptidylprolyl isomerase B
pRb: retinoblastoma protein
SALL2: Spalt‐like 2
SALL: Spalt‐like
SKOV3: SK‐ovary adenocarcinoma cells
SV40: simian virus 40
SWI/SNF: SwItch/sucrose nonfermenting
WT1: Wilms tumor 1
ZO‐2: zonula occludens‐2

## 1 Introduction

SALL2 belongs to the Spalt‐like family of transcription factors conserved from nematodes to humans (de Celis and Barrio, 2009; Sweetman and Munsterberg, 2006). The gene contains two alternative promoters (P1 and P2), which originate two protein isoforms, named SALL2‐E1 and SALL2‐E1A (Ma *et al*., 2001). These two isoforms differ in the first 25 amino acids (Ma *et al*., 2001), and those amino acids are particularly relevant as SALL2‐E1 contains a 12 amino acids conserved motif involved in transcriptional repression through its interaction with the NuRD complex (Lauberth and Rauchman, 2006). Both SALL2 isoforms contain several C_2_H_2_ zinc finger motifs along the structure, and several glutamine‐, serine‐, and proline‐rich regions, which are typically present in transcription factors (Hermosilla *et al*., 2017). E1 is restricted to certain tissues such as thymus, testis, and colon, while E1A has ubiquitous expression. Both isoforms are highly expressed in the brain (Kohlhase *et al*., 1996, 2000; Ma *et al*., 2001).

SALL2 has been associated with neurogenesis, neuronal differentiation, and eye development. Consequently, *SALL2/Sall2* deficiency associates with neural tube defects in mice, and with coloboma, a congenital eye disease in humans and mice (Böhm *et al*., 2008; Kelberman *et al*., 2014). Importantly, SALL2 is deregulated and/or mutated in various cancers, suggesting a role for SALL2 in the disease (Hermosilla *et al*., 2017). In this context, most findings suggest that SALL2 behaves as a tumor suppressor (Hermosilla *et al*., 2017; Sung and Yim, 2017). Like the retinoblastoma protein (pRb) and the p53 tumor suppressors (Yim and Park, 2005), SALL2 interacts with viral oncogenic proteins. These include the mouse polyomavirus large T antigen (Li *et al*., 2001) and the human papillomavirus type 16 E6 (HPV16 E6) protein (Parroche *et al*., 2011), but fails to interact with the large T antigen of simian virus 40 (SV40) (Li *et al*., 2004)

SALL2 has also been associated with induction of cellular quiescence in human fibroblasts (Liu *et al*., 2007) and with cellular apoptosis in mouse embryonic fibroblast and human leukemia cells exposed to genotoxic stress (Escobar *et al*., 2015). Clinical evidence showed loss of heterozygosity (LOH) at the *SALL2* locus in 30% of ovarian cancer patients (Bandera *et al*., 1997), and recent studies demonstrated that the P2 promoter of SALL2 is susceptible to silencing by methylation (Sung *et al*., 2013). This epigenetic modification was confirmed in the majority of primary tumors and correlated with negative SALL2 expression in ovarian carcinomas of various histological types. Other studies indicate that lost or reduced *SALL2* expression may be involved in leukemogenesis (Chai, 2011) and breast cancer (Liu *et al*., 2014; Zuo *et al*., 2017). However, SALL2 is found upregulated in Wilms tumor (Li *et al*., 2002), synovial sarcoma (Nielsen *et al*., 2003), and oral (Estilo *et al*., 2009) and testicular cancer (Alagaratnam *et al*., 2011), indicating that SALL2 role in cancer is yet controversial.

Evidence of the role of SALL2 in proliferation came mainly from overexpression experiments. Initial studies showed that forced expression of SALL2 in SKOV3 ovarian carcinoma cells inhibits DNA synthesis and increases p21^WAF/CIP^ mRNA and protein levels (Li *et al*., 2004). Similarly, a microarray analysis indicates that the cyclin‐dependent kinase 4 inhibitor A (p16^INK4a^) gene (*CDKN2A*) is upregulated in SALL2‐expressing versus SALL2‐null SKOV3 ovarian carcinoma cells (Wu *et al*., 2015). Together, these data suggest that SALL2 inhibits cell cycle progression at the G1‐ to S‐phase transition by upregulation of cyclin‐dependent kinase inhibitors. However, few direct transcriptional target genes of SALL2 related to proliferation have been identified.

To further investigate the role of SALL2 in cell proliferation, we used primary and immortalized mouse embryonic fibroblasts (MEFs) derived from previously characterized *Sall2*‐deficient mice (Sato *et al*., 2003). Our studies showed that SALL2 is highly expressed in mitotic cells. After cell division, SALL2 protein levels are slightly reduced and then are maintained constant during progression through G1. We demonstrated that SALL2 exerts a negative regulatory role in cell proliferation associated with the regulation of cell cycle progression. We identified a novel mechanism involving the transcriptional repression of G1‐S cyclins, *CCND1* and *CCNE1,* by SALL2. Accordingly, we observed inverse correlation between SALL2 and G1‐S cyclins levels in specific tissues, supporting their negative regulation by SALL2 *in vivo*. Considering that *Sall2* ^*−*/*−*^ MEFs displayed transformation properties and data from R2 platform show a negative correlation between *SALL2* and G1‐S cyclins mRNA expression in various cancers, our studies further support a tumor suppressor role for SALL2.

## 2 Materials and methods

### 2.1 Reagents

Propidium iodide, nocodazole (#M1404), SALL2 (#HPA004162) polyclonal antibody, protease inhibitor cocktail I (# P8340), phosphatase inhibitor cocktail II (P5726), and 5‐bromo‐2′‐deoxyuridine (# B5002) were purchased from Sigma‐Aldrich Chemicals (St. Louis, MO, USA). SALL2 antibody used for ChIP experiments was obtained from Bethyl Lab (Montgomery, TX, USA). Cyclin A (C‐19, #SC‐596) polyclonal antibody and cyclin B1 (GNS1, #SC‐245), cyclin D1 (DCS‐6, #SC‐20044), cyclin E1 (E‐4, #SC‐377100), p21 (F‐5, #6246), Myc (9E10, #SC‐40), and β‐actin (AC‐15, #SC‐69879) monoclonal antibodies were obtained from Santa Cruz Biotechnology (San Diego, CA, USA). The SV40 large T antigen expression pBSSVD2005 plasmid was a gift from David Ron (Addgene plasmid # 21826), the plasmid containing the *CCNE1* promoter was a gift from Bob Weinberg (Addgene plasmid # 8458) (Geng *et al*., 1996), and the *CCND1* promoter pGL3Basic was a gift from Frank McCormick (Addgene plasmid # 32726) (McCormick and Tetsu, 1999). pcDNA3‐SALL2 plasmid was described elsewhere (Escobar *et al*., 2015). Alexa Fluor 488‐conjugated phalloidin and Alexa Fluor 488‐conjugated goat anti‐rabbit secondary antibodies were purchased from Invitrogen (Carlsbad, CA, USA). Horseradish peroxidase‐conjugated secondary antibodies and Hoechst 33342 were from Bio‐Rad (Hercules, CA, USA).

### 2.2 Isolation of primary MEFs and genotyping

*Sall2* knockout mice (Sato *et al*., 2003) were obtained by collaboration with Dr. Ruichi Nishinakamura (Kumamoto University, MTA (2010) to RP, Universidad de Concepción). Mice were group‐housed under standard conditions with food and water available *ad libitum* and were maintained on a 12‐h light/dark cycle. Mice were fed with a standard chow diet (ProLab, LabDiet, St. Louis, MO, USA) containing no less than 5% crude fat and were treated in compliance with the US National Institutes of Health guidelines for animal care and use. Studies were reviewed and approved by the Animal Ethics Committee of the Chile's National Commission for Scientific and Technological Research (CONICYT, protocol for projects # 1110821 and # 1151031).

*Sall2* ^*+/+*^ and *Sall2* ^*−/−*^ fibroblasts were prepared from embryos at 13.5 days *postcoitum* as previously described (Escobar *et al*., 2015). Briefly, embryos, whose head and other red organs were removed, were smashed into pieces using a razor blade in a 100‐mm dish with 5 mL trypsin (GE Healthcare HyClone, Logan, UT, USA). The smashed embryo was incubated in trypsin for 15 min at 37 °C followed by dilution in 10 mL DMEM (GE Healthcare HyClone) by pipetting up and down. Cells were centrifuged and seeded in 100‐mm culture dishes (passage 0). MEFs were generated from independent embryos and routinely cultured as described below.

Mice were routinely genotyped by isolating tail DNA as previously reported (Escobar *et al*., 2015). One microliter of genomic DNA was used for PCR analysis. *Sall2* PCR was performed as previously (Escobar *et al*., 2015) with the following oligonucleotides: forward, 5′‐CACATTTCGTGGGCTACAAG‐3′, and reverse, 5′‐CTCAGAGCTGTTTTCCTGGG‐3,′ and Neo, 5′‐GCGTTGGCTACCCGTGATAT‐3′. The sizes of the PCR products are 188 bp for the wild‐type (WT) and 380 bp for the null mutant.

### 2.3 Cell culture

*Sall2* ^*+/+*^ and *Sall2* ^*−/−*^ primary and immortalized MEFs were cultured in DMEM supplemented with 10% heat‐inactivated fetal bovine serum (FBS, GE Healthcare HyClone), 1% glutamine (Invitrogen), and 0.5% penicillin/streptomycin (Invitrogen). Experiments with primary *Sall2* ^*+/+*^ and *Sall2* ^*−/−*^ MEFs were performed with early passages (passages 3–4). Human embryonic kidney epithelial HEK293 cells (American Type Culture Collection CRL‐1573™) used for promoter reporter assays and chromatin immunoprecipitation were cultured in DMEM supplemented with 10% FBS, 1% glutamine, and 0.5 % penicillin/streptomycin.

### 2.4 3T3 assays

Primary MEFs from passages 3–4 were seeded at 3 × 10^5^ cells/60 mm dish, cell numbers were determined after 3 days, and cells were reseeded for the next passage at the starting density. This protocol was repeated between 15 and 18 times.

### 2.5 MEFs immortalization

Primary *Sall2* ^*+/+*^ and *Sall2* ^*−/−*^ MEFs (passage 4) were immortalized using SV40 large T antigen based on modified protocol from Zhu *et al*. (1991). For transfection, we used Lipofectamine 2000 (Invitrogen) and 2 μg SV40 large T antigen expression vector. After cell transfection, we proceeded to select for low density. To complete the immortalization process, 5–6 post‐transfection passages were carried out.

### 2.6 CRISPR/Cas 9‐mediated gene targeting

HEK293 cells were electroporated with a vector encoding CRISPR/Cas9 coupled to Paprika‐RFP and harboring the following guide RNA against exon 2 of SALL2 5′ GGCTCCTTAGGCCAGACGGT 3′. Cas 9 and Paprika‐RFP genes are linked by the 2A oligopeptide sequence, allowing efficient production of the two proteins by ribosome skipping translation (Provost *et al*., 2007). After 16 h postelectroporation, the top 2% of the brightest cells were sorted by RFP channel and plated as individual clones. The clones were grown for 2 weeks, and western blot against SALL2 was performed in each clone for knockout identification. After selection of positive clones, genomic PCR and further sequencing confirmed CRISPR/Cas9 cut on the *SALL2* locus.

### 2.7 Proliferation assays

Primary MEFs were seeded at 2 × 10^5^ cells/35‐mm dish in triplicate, and cells were counted daily for 6 days. Media were replaced every second day. The immortalized *Sall2* ^*+/+*^ and *Sall2* ^*−/−*^ MEFs were seeded at 1 × 10^4^ cells/well in 6‐well dishes in triplicate, and cells were counted daily for 6 days.

### 2.8 Cell synchronization

Exponentially growing immortalized MEFs (iMEFs) were treated with 125 ng mL^−1^ nocodazole for 16 h. Except for flow cytometry (FACS) analyses where the whole cell population was collected to avoid morphological disruption, mitotic cell population was enriched by mechanic detachment (shake‐off) as described by Schorl and Sedivy (2007). Cells were released by a single rinse and subsequent incubation with fresh nocodazole‐free complete media. After nocodazole release, cells were harvested at selected times for western blot, qRT/PCR, and FACS analyses**.**

### 2.9 Flow cytometry

Approximately, 1.5 × 10^6^ iMEFs/100‐mm dish were seeded the day before synchronization. At the moment of harvest, cells were washed in phosphate‐buffered saline (PBS). Both culture media and PBS used for rinsing the cells were collected. After PBS wash, cells were detached in 0.25% trypsin and collected in the same tube. Following centrifugation, cells were washed again with PBS, suspended in 300 μL of ice‐cold PBS, and fixed by adding 700 μL of ice‐cold 70% ethanol drop wise to the sample while vortexing, and kept at 4 °C. After fixation, cells were washed twice with PBS and incubated with 0.4 mg mL^−1^ RNAse (Thermo Fisher Scientific, Waltham, MA, USA) in PBS at 37 °C during 40 min. Propidium iodide was added to a final concentration of 10 μg mL^−1^. Cells were filtered and analyzed for DNA content on a Becton Dickinson FACSCanto II. Cell population was quantified with modfit lt 5.0.9 software (Verity Software House, Inc., Topsham, ME, USA).

### 2.10 BrdU incorporation experiments

Cells were synchronized as described above. After mitotic detachment, floating cells were seeded on poly‐l‐lysine‐coated coverslips and grown in nocodazole‐free medium. Cells were cultured in 50 μm BrdU‐supplemented media for 45 min prior to fixation in cold‐fixing solution (70% ethanol, 15 mm glycine; pH 2.0). Cells were rinsed twice with PBS 1X and blocked in 3% BSA‐PBS 1X solution. Coverslips were incubated with 1:20 anti‐BrdU antibody or 1 : 100 anti‐cyclin D1 (M20, #SC‐718) in incubation buffer (Roche Applied Science, Indianapolis, IN, USA) for 40 min at 37 °C. Next, cells were incubated with 1 : 500 fluorophore‐conjugated secondary antibody (Molecular Probes, Invitrogen) in 1% BSA‐PBS 1X solution. Cells were PBS‐rinsed again, and nuclei were stained with Hoechst (Bio‐Rad).

### 2.11 Western blot analysis

Cells were lysed in 50 mm Tris/HCl pH 7.4, 200 mm NaCl, 2.5 mm MgCl_2_, 1% Triton X‐100, and 10% glycerol, supplemented with protease and phosphatase inhibitor cocktails. Proteins from cell lysates (50–70 μg total protein) were fractionated by SDS/PAGE and transferred overnight at 30 mA to PVDF membrane (Immobilon, Merck, Kenilworth, NJ, USA) using a wet transfer apparatus. The PVDF membranes were blocked for 1 h at room temperature in 5% nonfat milk in TBS‐T (TBS with 0.1% Tween) and incubated overnight with an appropriate dilution of primary antibody at 4 °C. After washing, the membranes were incubated with horseradish peroxidase‐conjugated secondary antibody (Bio‐Rad) diluted in 5% nonfat milk in TBS‐T for 1 h at room temperature. Immunolabeled proteins were visualized by ECL (Pierce, Thermo Scientific, Waltham, MA, USA). For the study of protein expression *in vivo*, proteins were extracted from several tissues (kidney, spleen, brain, cerebellum, and liver) of 6‐week‐old *Sall2* ^*+/+*^ and *Sall2* ^*−/−*^ mice. Tissues were lysed in 50 mm Tris/HCl pH 7.5, 150 mm NaCl, 2.5 mm MgCl_2,_ 1% NP‐40, 0.1% deoxycholate, and 10% glycerol, supplemented with protease and phosphatase inhibitor cocktails. Lysates were analyzed by western blotting as described above.

### 2.12 Focus formation assay

*Sall2* ^*+/+*^ and *Sall2* ^*−/−*^ iMEFs were seeded at 1 x 10^4^/well in 6‐well dishes and cultured in complete medium with 10% FBS, without splitting, for 14–21 days. Media were replaced every 2 days. Confluent monolayer cultures with foci were rinsed with PBS and stained with 4 mg mL^−1^ crystal violet in 10% methanol. Experiments were performed in triplicate.

### 2.13 Anchorage‐independent growth by soft agar assay

*Sall2* ^*+/+*^ and *Sall2* ^*−/−*^ iMEFs were seeded at 1.5 × 10^4^/60‐mm dish in 1.8 % Bacto agar in DMEM supplemented with 10% FBS. Every 2 days, 0.5 mL of growth media was applied to the surface of the agar to prevent it from drying out. After incubating for 4 weeks at 37 °C in the 5% CO_2_ incubator, colonies were counted. Experiments were performed in triplicate.

### 2.14 Transient transfections and reporter gene assays

To evaluate transcriptional activity of *CCNE1* and *CCND1* promoters, HEK293 cells were transiently co‐transfected with 0.5 μg of each promoter cyclin (CCNx‐luc), 0.125 μg of RSV‐β‐galactosidase (β‐Gal), and 1 μg of SALL2 (pcDNA3SALL2 E1 and pcDNA3SALL2 E1A**)** or control vector per well. After 24 h, the transfected cells were washed with PBS, lysed with reporter assay lysis buffer (Promega, Madison, WI, USA), and spun at 14 000 ×  g to pellet cell debris. The supernatant was then assayed for luciferase and β‐Gal activity using the manufacturer's suggested protocols. Luminescent reporter activity was measured using a Luminometer (Victor3; Perkin‐Elmer, Waltham, MA, USA). All transfections were normalized to β‐Gal activity and performed in triplicate. Luciferase values were expressed as fold induction relative to luciferase.

### 2.15 ChIP assay

The assay was carried out as previously (Henriquez *et al*., 2011) with the following modifications: HEK293 cells (2 × 10^6^ cells/100 mm plate) were transfected with pFLAG‐CMV2‐SALL2E1 or pFLAG‐CMV2‐SALL2E1A. To shear DNA, nuclei were sonicated in 300 μL of sonication buffer using a Misonix sonicator (model 3000) (16 times, 15 s on/20 s off each time, 6 W potency), obtaining DNA lengths between 300 and 500 bp. Immunoprecipitations were carried out overnight at 4 °C using 5 μg anti‐SALL2 (Bethyl) or 5 μg normal mouse IgG antibodies, and 40 μg of chromatin. DNA was analyzed by real‐time PCR directed to *SALL2*‐specific proximal regions of *CCNE1* (−70/−242) and *CCND1* (−22/−201) promoters. Primer sequences were forward, 5′ CTGATTCCCCGTCCCTGCG 3′, and reverse, 5′ GACATTTAAAATCCCTGCGCGC 3′, for *CCCNE1;* forward, 5′ TCTATGAAAACCGGACTACAGG 3′, and reverse, 5′ AAAGATCAAAGCCCGGCAGA 3′, for *CCND1*. In addition, a previously reported region of *Pmaip1* promoter (−869/−756) (Escobar *et al*., 2015) was used as negative control of SALL2 binding. Primer sequences for this unrelated region (URR) were forward, 5′ TGAAGCGGCTCTCAGTAACC 3′, and reverse, 5′ AGCTACCTGGGAACGTGAAA 3′. All PCRs (KAPA SYBR FAST qPCR; Kappa Biosystems, Wilmington, MA, USA) contained 1 μL of input and 3 μL of IP samples.

### 2.16 Real‐time quantitative reverse transcription/PCR

Total RNA was extracted from cells with TRIzol reagent (Thermo Fisher Scientific) according to the manufacturer's instructions. RNA was treated with Turbo DNase (Invitrogen Ambion, Thermo Fisher Scientific) to eliminate any residual DNA from the preparation. Total RNA (1 μg) was reverse‐transcribed using the Maloney murine leukemia virus reverse transcriptase (Invitrogen) and 0.25 μg of Anchored Oligo(dT) 20 Primer (Invitrogen; 12577‐011). To control specificity of the amplified product, a melting curve analysis was carried out. No amplification of unspecific product was observed. Amplification of cyclophilin B (*Ppib*) was carried out for each sample as an endogenous control. Primer sequences were forward, 5′ GATCTCCTCCGCAGTCTGG 3′, and reverse, 5′ ACACAATGGGTATCCGGTCT 3′, for mouse *Sall2 E1A*; forward, 5′AACGGAGACCCCAACAGTTA 3′, and reverse, 5′ TGGGTCAGTGCAACATGAGT 3′, for mouse *Sall2E1*; forward, 5′ GTGCTGGGAATGCAAGCCATATCT 3′, and reverse, 5′ AAGCGGCTGGAAATGGCTTAGT 3′, for *Ccne1*; forward, 5′ AGGAAGCGGTCCAGGTAGTT 3′, and reverse, 5′ AGTGCGTGCAGAAGGAGATT 3′, for *Ccnd1;* forward, 5′ TTGTGGCCTTAGCTACAGGA 3′, and reverse, 5′ GCTCACCGTAGATGCTCTTT3′, for *Ppib*.

The relative expression of the *Ccne1, Ccnd1,* and *Sall2* genes was calculated using the standard curve method, and all mRNA expressions were relative to *Ppib*.

## 3 Data accessibility

Publicly available databases from R2: Genomics Analysis and Visualization Platform (http://r2.amc.nl) were used as materials for this study.

## 4 Results

### 4.1 Enhanced cell proliferation of primary *Sall2*‐deficient MEFs

**Figure 1:**
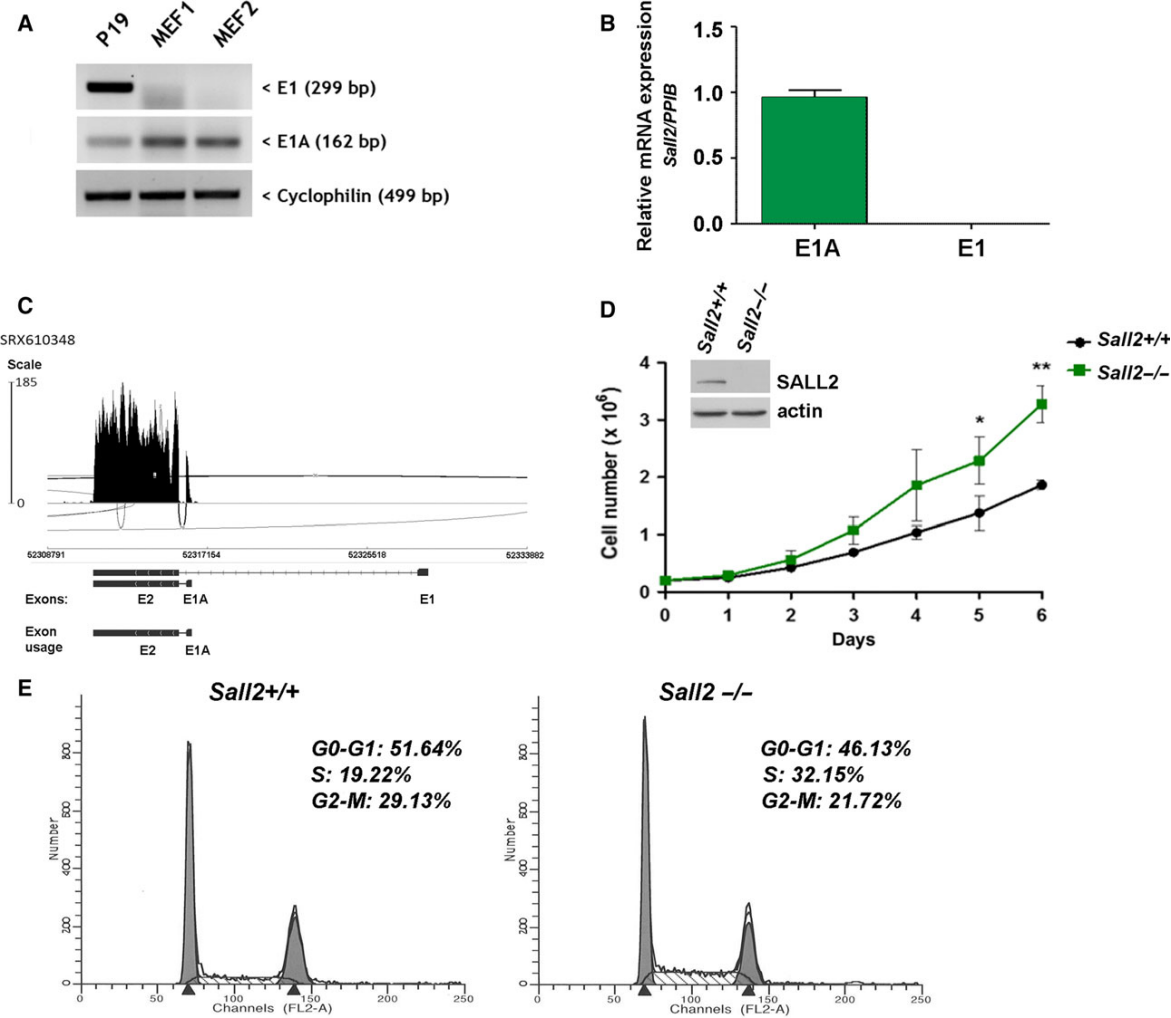
Increased proliferation of *Sall2*‐deficient cells. (A,B) Expression of *Sall2* mRNA isoforms was evaluated in *Sall2* wild‐type (*Sall2* ^*+/+*^) MEFs by RT/PCR (A) and qPCR (B) using *Ppib* as normalizer. P19 cells were used as positive control for SALL2 isoforms expression. (C) Sashimi plot (http://software.broadinstitute.org/software/igv/Sashimi) for alternatively spliced exon and flanking exon of *Sall2* from deep RNA‐sequencing data of MEFs (SRX610348). Per‐base expression is plotted on *y*‐axis of Sashimi plot, genomic coordinates on *x*‐axis, and mRNA isoforms are represented on the bottom (exons in black, introns as lines). (D) Proliferation curves of *Sall2* ^−/−^ and *Sall2* ^*+/+*^ MEFs. Equal number of MEFs (passage 3) was plated in triplicate and counted every day for 6 days. Data are expressed as mean ± SD from three independent experiments. *P ≤ 0.05, **P ≤ 0.01, Student's *t‐test*. (E) Cell cycle analysis of *Sall2* ^−/−^ and *Sall2* ^*+/+*^ MEFs by flow cytometry. Asynchronous cells (passage 4) were collected, fixed, and stained with propidium iodide (PI) before cell cycle analysis. G0‐G1, S and G2‐M populations are indicated as percentages of the whole population. Figure is representative of two independent experiments of isogenic MEFs performed in triplicate.

To further investigate the role of SALL2 in cell proliferation, we used primary *Sall2*‐null mouse embryo fibroblasts (MEFs) derived from previously characterized *Sall2*‐deficient (*Sall2* ^*−/−*^ *)* mice (Sato *et al*., 2003). Because *Sall2* gene could rise two isoforms of similar molecular weight (Ma *et al*., 2001), we first evaluated isoforms’ expression in wild‐type (*Sall2* ^*+/+*^) MEFs by RT/PCR. P19 mouse cells, which express both isoforms, were used as positive control. We found that *Sall2*‐*E1A* (162‐bp band) is the predominant isoform in MEFs under normal cell culture conditions, while *Sall2*‐*E1* isoform (299‐bp band) is barely detected (Fig. 1A). Quantitative RT/PCR (qPCR) also showed that *Sall2 E1A* is abundant in MEFs, while *E1* was undetectable (Fig. 1B). The low/absent expression of E1 isoform was additionally confirmed by analyzing transcriptome sequencing of MEFs available from public datasets (http://www.ebi.ac.uk/ena, SRX610348). A Sashimi plot depicts preferential usage of isoform E1A from E1 in MEFs (Fig. 1C).

We then compared proliferation rate of primary *Sall2* ^*+/+*^ and *Sall2* ^*−/−*^ MEFs. Under normal growth conditions, *Sall2* ^*−/−*^ cells proliferate significantly faster than *Sall2* ^*+/+*^ cells (Fig. 1D). Similar results were obtained from three independent isogenic *Sall2* ^*+/+*^ and *Sall2* ^*−/−*^ MEFs cultures (data not shown). Furthermore, flow cytometry data analysis indicated that asynchronous *Sall2* ^*−/−*^ MEFs present higher proportion of cells in S phase (32.2% vs. 19.2%) and lower proportion of cells in G2‐M (21.7% vs. 29.1%) than *Sall2* ^*+/+*^ cells (Fig. 1E).

Because of the limited lifespan of primary MEFs, we attempted to generate immortal MEFs using the 3T3 protocol (Xu, 2005); however, *Sall2* deficiency did not result in spontaneous immortalization of fibroblasts. In fact, our study showed that *Sall2* ^−/−^ MEFs enter senescence and have a lifespan similar to *Sall2* ^*+/+*^ MEFs (Fig. S1A). At passage 8, we noticed that both *Sall2* ^−/−^ and *Sall2* ^*+/+*^ MEFs present the typical senescence phenotype consisting of enlarged cell with an increased SA‐β‐galactosidase activity (Fig. S1B). Thus, considering that SV40 large T antigen fails to interact with SALL2 (Li *et al*., 2004), we immortalized MEFs using this antigen‐mediated transformation (Zhu *et al*., 1991). Primary cells were transfected with SV40 T antigen, and after five passages using cell density selection, immortal *Sall2* ^*+/+*^ and *Sall2* ^−/−^ MEFs were obtained. There were no obvious phenotypic differences between *Sall2* ^*+/+*^ and *Sall2* ^−/−^ iMEFs grown for 24 h in complete medium (Fig. S2A,B). According to previous data on the role of SALL2 in the proliferation of cancer cells (Li *et al*., 2004), and with our data on primary MEFs (Fig. 1), *Sall2* ^−/−^ iMEFs also showed higher rate of proliferation compared to the *Sall2* ^*+/+*^ counterpart (Fig. S2C). Together, these results indicate that *Sall2‐*deficiency increases cell proliferation and suggest that SALL2 E1A negatively regulates cell proliferation in embryonic fibroblasts.

### 4.2 *Sall2* deficiency accelerates postmitotic progression into G1 and S phases

**Figure 2:**
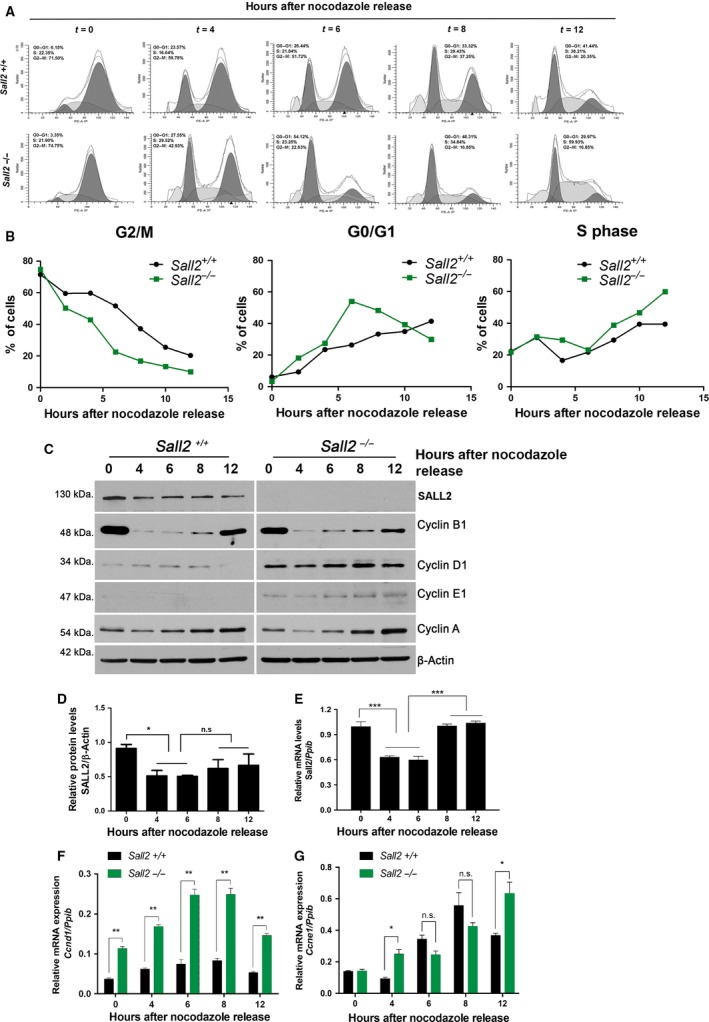
*Sall2* deficiency accelerates G1‐/S‐phase transition and correlates with increased levels of cyclins D1 and E1. *Sall2* ^*+/+*^ and *Sall2* ^−/−^ iMEFs were synchronized at G2‐M by nocodazole as described in materials and methods, and then released into the cell cycle. (A) Cell cycle analysis of *Sall2* ^*+/+*^ and *Sall2* ^−/−^ iMEFs by flow cytometry. The graphs show representative cell cycle profiles of each iMEFs over time (hours) after release from nocodazole. (B) Progression of *Sall2* ^*+/+*^ iMEFs through the cell cycle was compared with that of *Sall2* ^−/−^ iMEFs every 2 h for a total of 12 h. Data are presented as the percentage of cells at G2‐M, G1, and S phases over time after nocodazole release and are representative of four independent experiments. (C) *Sall2* ^*+/+*^ and *Sall2* ^−/−^ iMEFs were harvested at various times after release to evaluate SALL2 protein expression and compare the expression of the indicated cyclins by western blot. β‐actin was used as normalizer. Figure is representative of four independent experiments. (D) Graph shows relative SALL2 protein level in *Sall2* ^*+/+*^ iMEFs after various hours from nocodazole release. SALL2 protein level was determined by densitometric analysis and normalized to the corresponding β‐actin level. Data are expressed as mean ± SD from four independent experiments. *P ≤ 0.05, one‐way ANOVA; n.s, not significant. (E) *Sall2* mRNA expression was evaluated by qPCR analysis using *Ppib* as normalizer. Data are expressed as mean ± SD from three independent experiments performed in triplicate. ***P ≤ 0.001, one‐way ANOVA. (F,G) RNA was isolated from *Sall2* ^*+/+*^ and *Sall2* ^−/−^ iMEFs and mRNA levels of *Ccnd1* (F) and *Ccne1* (G) were measured by qPCR. Numbers are relative to *Ppib*. Data are expressed as mean ± SD from three independent experiments performed in triplicate. *P ≤ 0.05; **P ≤ 0.01, Student's *t*‐test; n.s, not significant.

Because of the proliferative advantage of *Sall2‐*deficient MEFs, we investigated the expression profile of SALL2 during cell cycle progression. iMEFs were synchronized in mitosis using nocodazole. Mitotic iMEFs were harvested and then reseeded in complete medium for cell cycle re‐entry into G1. Mitotic synchronization and progression into G1 and S phases were monitored by FACS analysis and expression of specific cell cycle markers (cyclins B, D, E, and A). About 80% of *Sall2* ^*+/+*^ and *Sall2* ^−/−^ iMEFs are arrested in mitosis after nocodazole treatment (*t* = 0), consistent with the high levels of cyclin B1 protein (M‐phase marker) (Fig. 2A–C). After nocodazole release (2–6 h), the percentage of mitotic *Sall2* ^−/−^ *and Sall2* ^*+/+*^ iMEFs progressively decreases to about 20% and 50%, while the percentage of cells in G1 increases to about 50% and 20%, respectively, showing accelerated postmitotic progression of *Sall2* ^−/−^ iMEF into G1 phase. Consequently, progression beyond G1/S phase transition is evident in *Sall2* ^−/−^ iMEFs by 4–12 h. At 12 h, the fraction of *Sall2* ^−/−^ cells in S phase increases to 60%, whereas *Sall2* ^+/+^ iMEFs only reach to 40% (Fig. 2A,B). The increase in the percentage of *Sall2* ^*−/−*^ iMEFs in S phase was also confirmed by BrdU incorporation experiments (Fig. S3).

Interestingly, SALL2 protein is highly expressed in mitotic *Sall2* ^+/+^ iMEFs (Fig. 2C,D). After cell division (4 h), SALL2 protein levels are slightly reduced and then are maintained constant between 6 and 12 h (Fig. 2C,D). Quantification of *Sall2* mRNA showed a similar decrease between 4 and 6 h, but it significantly increases over time (8–12 h) (Fig. 2E), suggesting differences between the behavior of SALL2 protein and mRNA at latter times of nocodazole release. Together, these results suggest that SALL2 regulates postmitotic progression into G1 as well as progression from G1 to S phases.

### 4.3 Cyclin D1 and E1 transcripts are increased in *Sall2‐deficient* cells

To understand the mechanism underlying SALL2 regulatory role in cell cycle progression, we compared the expression of specific cell cycle markers associated with the control of postmitotic progression into G1 and S phases by western blot (Fig. 2C). Whereas not obvious difference for the expression of cyclin B1 (mitotic marker) and cyclin A (S‐phase marker) was noticed between *Sall2* ^*+/+*^ and *Sall2* ^−/−^ iMEFs, a consistent upregulation of cyclin D1 (G1 phase) and cyclin E1 (S phase entry) was evident in *Sall2* ^−/−^ compared to *Sall2* ^*+/+*^ iMEFs (Fig. 2C).

Considering that protein expression of cyclins during cell cycle is regulated at transcriptional level (Klein and Assoian, 2008; Möröy and Geisen, 2004; Suryadinata *et al*., 2010), we evaluated changes in mRNA levels of cyclins D1 (*Ccnd1*) and E1 (*Ccne1*) during postmitotic progression through G1 and S phase, associated with the expression of SALL2. In agreement with protein levels of cyclins D1 and E1 in both genotypes, the levels of *Ccnd1* and *Ccne1* mRNA were significantly higher in *Sall2* ^−/−^ iMEFs compared to those in *Sall2* ^+/+^ iMEFs (Fig. 2F,G), suggesting that SALL2 could regulate expression at the transcriptional level of key factors (cyclins D1 and E1) controlling G1‐/S‐phase transition and promoting entry into S phase. These results indicate that accelerated postmitotic progression of *Sall2* ^−/−^ iMEFs into G1/S phases may be explained by a functional disruption of SALL2 on transcriptional repression of cyclins D1 and E1.

To confirm SALL2 regulation of cyclins expression, we approached rescue of SALL2 expression in *Sall2*‐deficient cells. Because of extremely low efficiency of transfection of iMEFs, we used HEK293 cells. We knocked out *SALL2* using CRISPR/Cas 9‐mediated gene targeting and then rescued SALL2 expression. Similar to the *Sall2* ^−/−^ MEFs, HEK‐SALL2KO cells showed upregulation of cyclin D1 levels (Fig. S4A,B). On the other hand, rescue of SALL2 significantly decreased the levels of cyclin D1 (Fig. S4C,D). The levels of cyclin E1 were already high at time 0 and were maintained constant after nocodazole release. We were unable to detect any significant difference in the levels of cyclin E1 between the SALL2WT and SALL2KO cells (Fig. S4A,B), or by the rescue of SALL2 (Fig. S4C,D). This later result suggests that cyclin E1 protein expression is mainly controlled by other factors in these cells. Altogether, these results suggest a regulatory role for SALL2 in cell cycle progression by downregulating the expression of G1 cyclins in both mouse fibroblast and human HEK293 cells.

### 4.4 SALL2 transcriptionally regulates G1‐S cyclins

**Figure 3:**
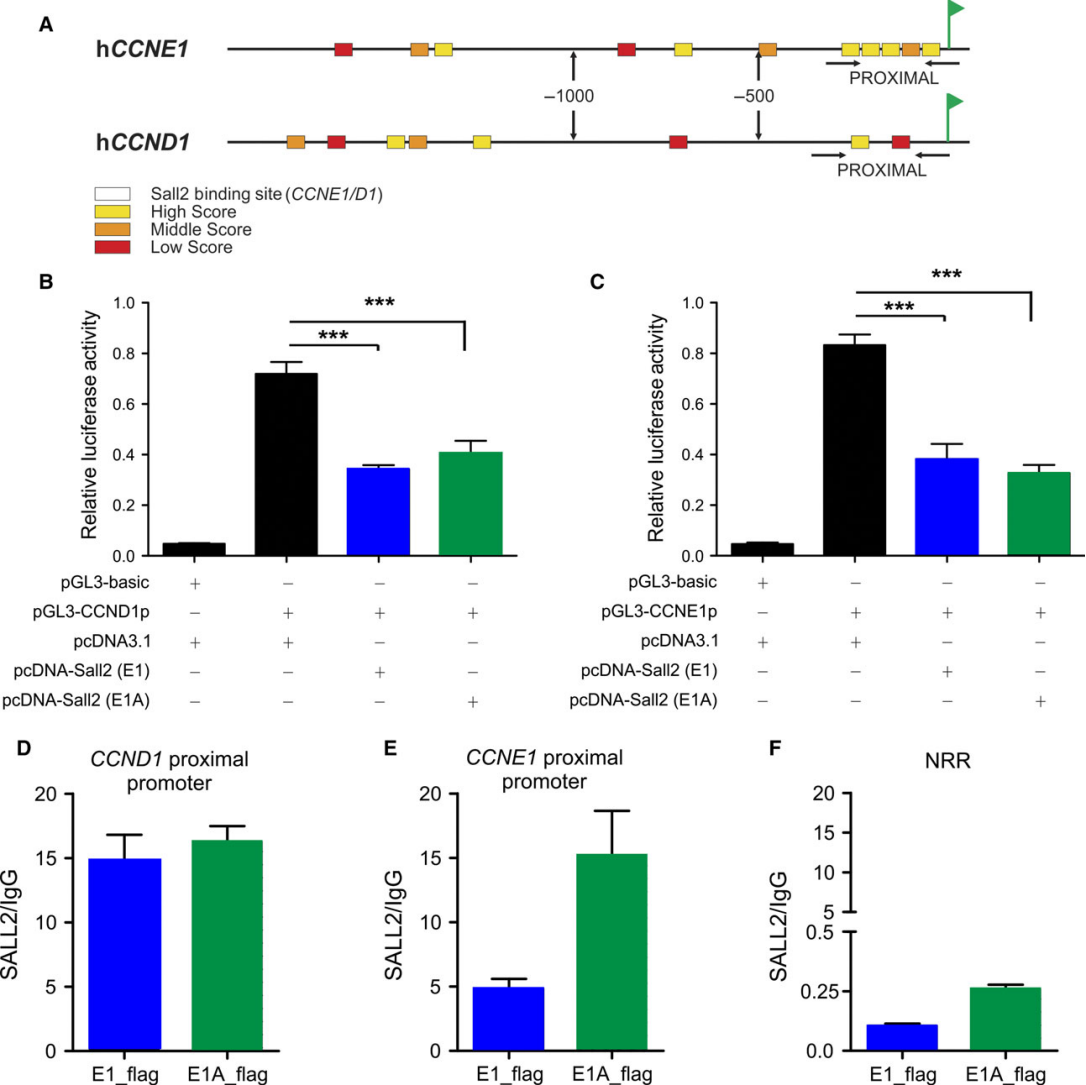
SALL2 binds and represses *CCND1* and *CCNE1* promoters. Bioinformatic analyses of cyclin promoters to identify putative SALL2 sites were performed using a previously reported binding site matrix (consensus sequence GGG(T/C)GGG) (Gu *et al*., 2011) in Transcriptional Regulatory Element Database (TRED) (https://cb.utdallas.edu/cgi-bin/TRED/tred.cgi?process=home) (Jiang *et al*., 2007). Sequences analyzed [−2000 bp from transcription start site (+1)] were obtained from Eukaryotic Promoter Database (EPD) and included mouse *Ccnd1* (NM 007631), mouse *Ccne1* (NM 007633), human *CCND1* (NM 053056), and human *CCNE1* (NM 001238). A. Schematic representation of human *CCND1* (NM 053056) and *CCNE1* (NM 001238) gene promoters. SALL2 putative binding sites are represented by square symbols. Putative SALL2 binding sites are classified as high, middle, and low scores according to their identity with the SALL2 consensus matrix (Gu *et al*., 2011) The transcription start site (+1) is represented by a flag. (B,C) Repression of *CCND1* (B) and *CCNE1* (C) promoters’ activities by SALL2. Transient co‐transfections of pGL3‐*CCND1* or pGL3*‐CCNE1* reporter with or without SALL2 E1 (blue bars) (or E1A, green bars) into HEK293 cells were performed as described under ‘Materials and Methods’. Luciferase activity was measured from cell lysates and normalized to β‐galactosidase activity, and promoter activity was expressed as relative luciferase units (R.L.U). pGL3 vector served as control. Data are expressed as mean ± SD from three independent experiments performed in triplicate. ***P ≤ 0.001, one‐way ANOVA. (D–F) HEK293 cells were transfected with pcDNA3‐SALL2 (E1, blue) or pcDNA3‐SALL2 (E1A, green) vector. Chromatin was immunoprecipitated 24 h after transfection using SALL2 antibody or normal rabbit IgG (control antibody), and specific genomic regions in the human *CCND1* (D)*, CCNE1* (E), proximal promoters, and a nonrelated promoter region (NRR) were analyzed by real‐time PCR. Graphs show quantification of the amplified DNA for each immunoprecipitation relative to IgG. Results are representative of two assays performed in triplicate.

To investigate whether SALL2 transcriptionally regulates cyclins D1 and E1, we initially performed bioinformatic analyses of mouse and human cyclin promoters to identify putative SALL2 binding sites (Fig. 3), using a previously reported binding site matrix (consensus sequence GGG(T/C)GGG) (Gu *et al*., 2011) in Transcriptional Regulatory Element Database (TRED) (https://cb.utdallas.edu/cgi-bin/TRED/tred.cgi?process=home) (Jiang *et al*., 2007). Conservation analysis using CLUSTAL‐O indicates 64% (*CCND1*) and 56% (*CCNE1*) identities between mouse and human promoters [−2000 bp from transcription start site (+1)], with the highest conservation in the proximal promoter region (−500 bp to +1). Identity between species in the proximal region is 69% for pCCND1 and 63% for pCCNE (Fig. S5). Thus, we focused on SALL2 binding sites at the proximal promoter because those could allow the binding of SALL2 protein to interfere with the transcriptional start site. Figure 3A shows a schematic representation of human promoters. Two putative SALL2 sites are present in *CCND1* promoter at positions −76 and −198 bp from transcription start site, while six putative sites are present in the proximal region of *CCNE1* promoter at positions −57, −68, −123, −140, −152, and −492 bp from transcription start site (+1) (Fig. S5).

Next, we evaluated responsiveness of human cyclin promoters to SALL2 using reporters previously described: hCCND1 (McCormick and Tetsu, 1999) and hCCNE1 (Geng *et al*., 1996). Expression of SALL2 (E1 or E1A isoform) significantly decreased the activity of *CCND1* and *CCNE1* promoters (Fig. 3B,C), indicating that both SALL2 isoforms repress *CCND1* and *CCNE1* promoter activity.

Finally, to demonstrate *in vivo* the interaction of SALL2 with *CCND1* and *CCNE1* promoters, we performed chromatin immunoprecipitation (ChIP) assays in HEK293 cells transfected with SALL2E1 or SALL2E1A. Figure 3D–E shows that both SALL2 isoforms bind to the *CCND1* and *CCNE1* proximal promoters. In contrast, no binding of SALL2 is observed to a nonrelated promoter region (Fig. 3F; NRR). Together, these results demonstrated that SALL2 binds to and regulates promoters of cyclins controlling cell cycle progression from G1 to S phase.

### 4.5 SALL2 and G1‐S cyclins’ expression inversely correlate *in vivo*

**Figure 4:**
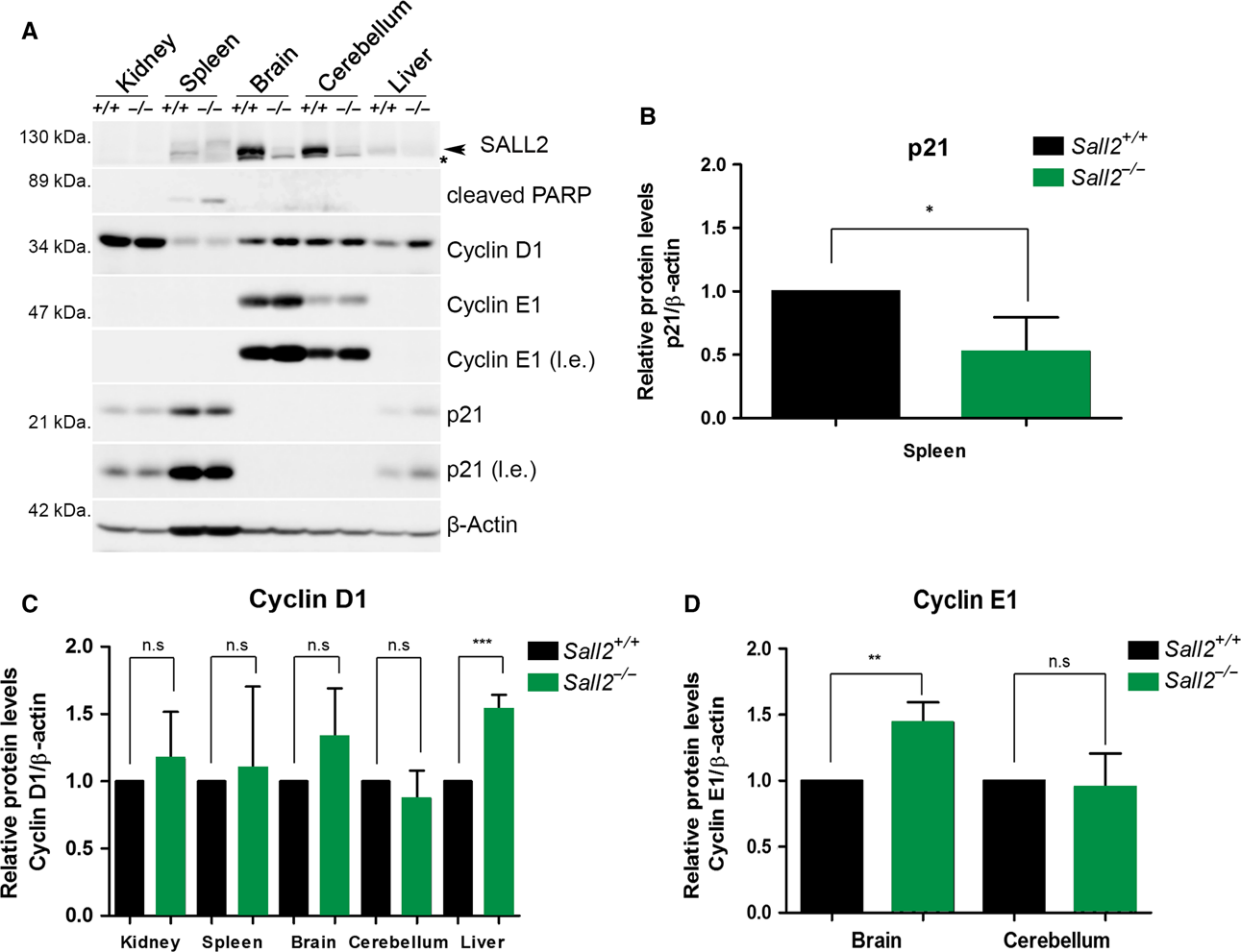
Increased expression of cyclins D1/E1 in tissues from *Sall2‐*deficient mice. Tissues from 6‐ to 8‐week‐old *Sall2* ^*+/+*^ and *Sall2* ^−/−^ mice were isolated and lysed to evaluate SALL2, cyclin D1 and cyclin E1 levels by western blot analysis (A). Representative western blot of tissues analyzed. An arrow indicates SALL2, and the asterisk corresponds to nonspecific band. p21—a protein positively regulated by SALL2 (Li *et al*., 2004)—was used as positive control; β‐actin was used as normalizer. l.e; long exposure. (B–D) Densitometric data from western blots of p21, cyclins D1 and E1 from five isogenic mouse/genotype. Data are expressed as mean ± SD from five independent mouse tissues per genotype. *P ≤ 0.05, **P ≤ 0.01, ***P ≤ 0.001, Student′s *t*–test; n.s, nonsignificant.

It has been reported that in adult organism, *Sall2* mRNA is highly expressed in the brain and to a lesser extent in heart, kidney, lung, pancreas, and ovary (Kohlhase *et al*., 1996, 2000; Ma *et al*., 2001). To evaluate the significance of SALL2‐dependent regulation of G1‐S cyclins *in vivo,* we compared the expression of cyclin D1 and E1 in several tissues from 6‐ to 8‐week‐old isogenic *Sall2* ^*+/+*^ and *Sall2* ^−/−^ mice by western blot and densitometric analysis. Figure 4 shows that SALL2 levels vary between tissues. Consistent with previous reports, the high levels of SALL2 were found in the brain and cerebellum, while it was poorly expressed in other tissues. As positive control of a SALL2‐dependent target, we evaluated p21 expression (Li *et al*., 2004). We noticed that only the spleen showed the expected positive correlation between p21 expression and the *Sall2* genotype (Fig. 4A,B). Consistent with a negative regulation of cyclins by SALL2, the levels of cyclin D1 were significantly upregulated in the liver (Fig. 4A,C) and the levels of cyclin E1 were significantly upregulated in the brain of *Sall2* ^−/−^ mice (Fig. 4A,D). No significant inverse correlation was found in other tissues analyzed, suggesting that SALL2‐dependent transcriptional regulation of cyclins D1 and E1 is tissue‐specific.

### 4.6 *Sall2*‐deficient iMEFs possess transformation capability

**Figure 5:**
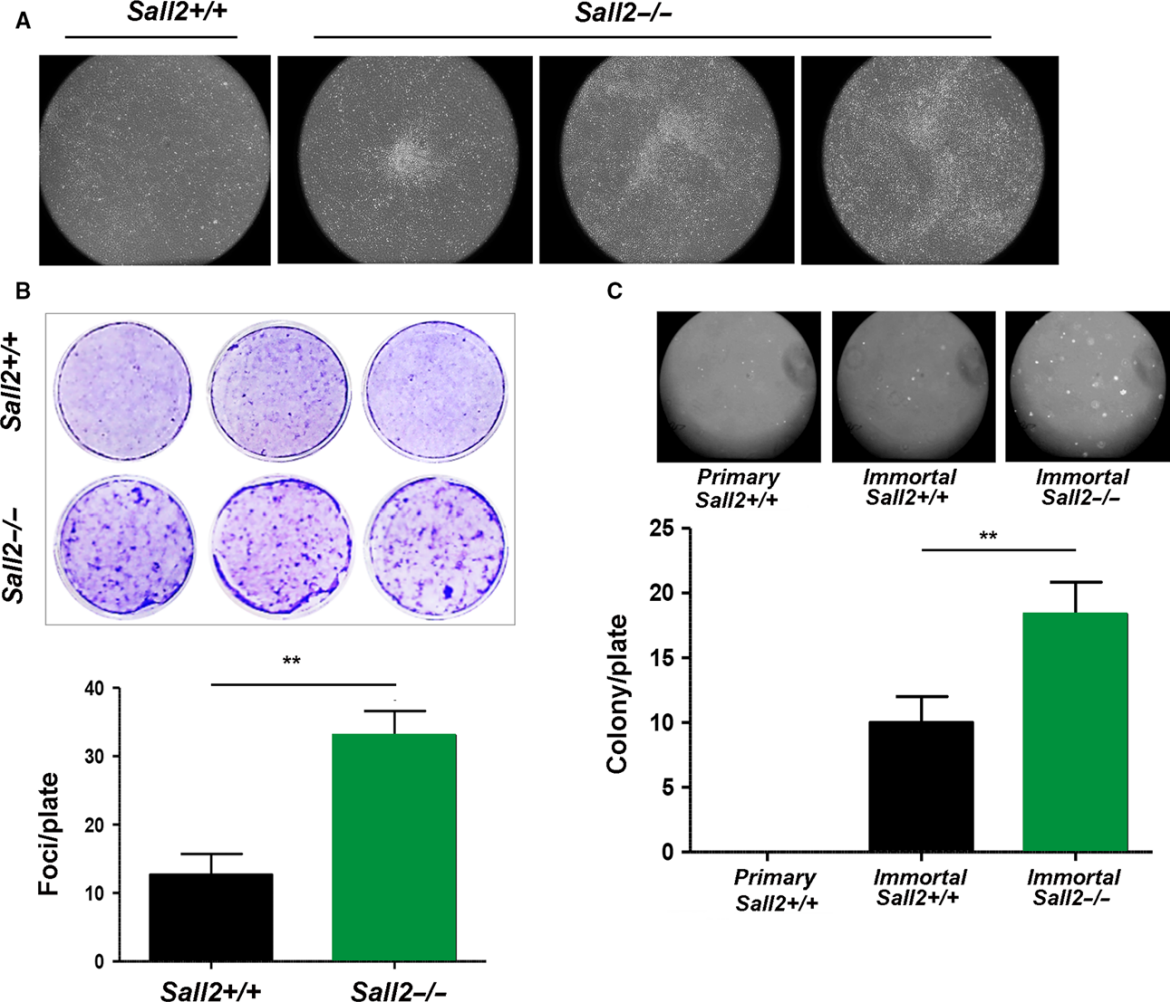
Immortalized *Sall2*‐deficient cells possess transforming ability. (A,B) Foci formation assay. iMEFs were grown in regular culture medium for 12–18 days prior to staining with crystal violet. (A) Microscopic visualization of three individual focus from *Sall2* ^−/−^ iMEFs photographed at 4× magnification. The appearance of the *Sall2* ^*+/+*^ iMEFs culture (left) is shown for comparison.(B) Top, crystal violet staining of *Sall2* ^*+/+*^ and *Sall2* ^−/−^ iMEFs. Bottom, quantification of number of foci per plate. Results are representative of three independent experiments performed in triplicate (**P ≤ 0.01, Student's *t*‐test). (C) *Sall2* ^−/−^ MEFs showed increased anchorage‐independent growth. *Sall2* ^*+/+*^ and *Sall2* ^−/−^ iMEFs were grown in soft agar for 3–4 weeks. Top, colonies were photographed at 4× magnification. Primary *Sall2* ^*+/+*^ MEFs were used as negative control. Bottom, quantification of number of colonies per plate. Results are representative of three independent experiments performed in triplicate (**P ≤ 0.01, Student's *t*‐test).

Cyclin D1 and/or E1 overexpression has been associated with cancer (Hwang and Clurman, 2005; Kim and Diehl, 2009; Möröy and Geisen, 2004; Qie and Diehl, 2016). Because of the increased growth rate and expression of cyclins D1 and E1 of *Sall2* ^−/−^ iMEFs, we evaluated whether these cells present enhanced transformation properties. Loss of contact inhibition was measured by focus‐forming assay. We detected transforming foci in the *Sall2* ^−/−^ iMEFs within 10–12 days of culture, but not in the *Sall2* ^*+/+*^ iMEFs. Figure 5A shows three representative colonies (focus) of morphologically transformed *Sall2* ^−/−^ iMEFs in relation to the *Sall2* ^*+/+*^ phenotype. Transformed cells were highly retractile and grew in irregular patterns with occasional balls or stellate patterns, particularly after prolonged incubation. After 16–18 days of culture, cells were fixed and stained to score the number of foci. *Sall2* ^−/−^ iMEFs presented an average of 33 foci/plate versus the 10 foci/plate observed in *Sall2* ^*+/+*^ iMEFs (Fig. 5B). We also performed soft agar colony formation assay to monitor anchorage‐independent growth. Primary *Sall2* ^*+/+*^ MEFs were used as negative control, and colony formation was compared between *Sall2* ^−/−^ and *Sall2* ^*+/+*^ iMEFs. *Sall2* ^*+/+*^ iMEFs grew few colonies, while *Sall2* ^−/−^ iMEFs significantly increased anchorage‐independent growth (Fig. 5C). These results confirm the malignant transformation properties of *Sall2‐*deficient cells and support a tumor suppressor role for SALL2.

### 4.7 *SALL2* and *CCND1/E1* genes’ expression inversely correlate in cancer

To assess correlation between *SALL2* and *CCND1/E1* genes’ expression in cancer, we used R2: Genomic Analysis and Visualization Platform (http://r2.amc.nl). The R2 platform allows analysis of multiple gene expression microarrays from various pathological conditions. Datasets from different types of cancer were used to investigate correlation between *SALL2* and *CCND1/E1* genes’ expression (Table S1). Tumor types were selected based on previous studies reporting deregulation of SALL2 in cancer (Liu *et al*., 2014; Ma *et al*., 2001). In addition, we selected studies performed in samples from patients without chemotherapy treatment.

**Figure 6:**
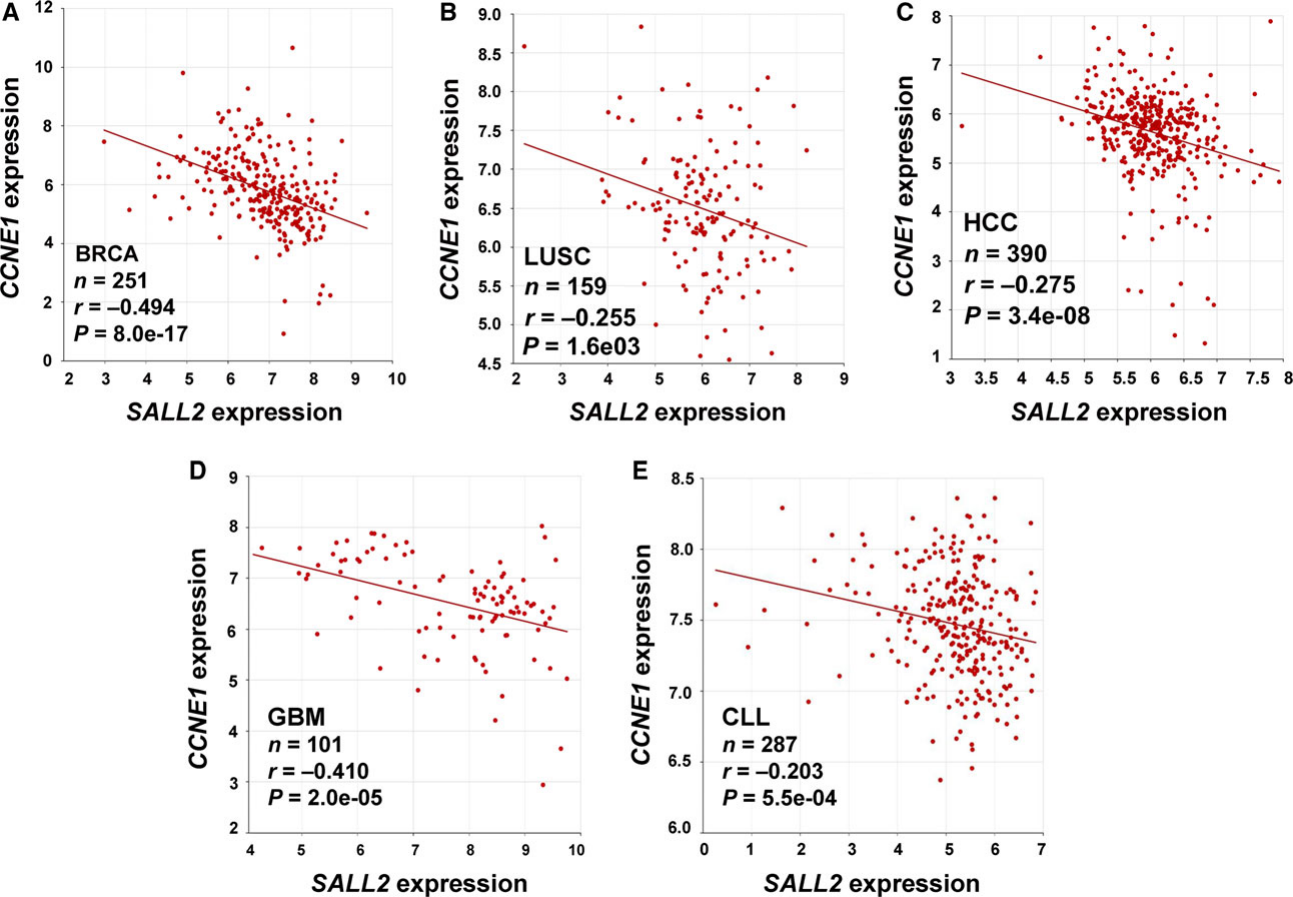
Correlation between *SALL2* and *CCND1/CCNE1* expression in cancer. Scatter plots of *SALL2* by *CCND1* and *CCNE1* were generated using publicly available databases and software's from R2: Genomics Analysis and Visualization Platform (http://r2.amc.nl). Pearson's correlation coefficients (*r*) and associated *P* values (p) were calculated using default HugoOnce algorithm and ANOVA statistical test. (A) BRCA, breast invasive carcinoma. (B) LUSC, lung squamous cell carcinoma, and (C) HCC, human colorectal cancer. (D) GBM, glioblastoma multiforme, (E) CLL, chronic lymphocytic leukemia, *n* = number of samples.

The microarray analyses showed significant inverse correlation between *SALL2* and *CCNE1* mRNA expression in various cancers, including glioblastoma, lymphoma, cervix, pancreas, breast, colon, and lung cancer (Table S1). As an example, Fig. 6 shows a representative graph from breast (A), lung (B), colon (C), glioblastoma (D), and lymphoma (E) cancer studies. Noteworthy, in breast cancer, four independent studies showed inverse correlations of similar magnitude, with Pearson's coefficients (*r*) ranging from −0.322 (GSE2109) to −0.494 (GSE3494) and *P* values ranging from 1.3e‐09 to 8.0e‐17 (Table S1). However, the correlation between *SALL2* and *CCND1* expression is puzzling. Although there is an inverse correlation in endometrial cancer (Table S1, GSE11869), tumor bladder (Table S1, GSE3167), pancreatic (Table S1, GSE17891), and colon cancer (Table S1, GSE41258), a positive correlation is found in breast cancer (Table S1, GSE3494, GSE2109, GSE21653, GSE2034) and sarcoma (Table S1; GSE17679). In some cancer studies (Table S1; GSE4536, GSE17891, GSE41258) *SALL2* levels inversely correlated with the expression levels of both *CCNE*1 and *CCND1*. Altogether, the R2 data analysis showed more consistent inverse correlation between *SALL2* and *CCNE1* expression in breast cancer tissues. Likely, SALL2 regulation of cyclin D1 and E1 expression is genetic context‐dependent, which could explain the observed inverse correlations in only a subset of cancers.

## 5 Discussion

Increasing studies indicate that SALL2 plays a role in cancer. Because it is downregulated in ovarian cancer as well as in other cancer types, SALL2 has been proposed as a tumor suppressor. However, SALL2 is also upregulated in some cancers and was recently identified as a key factor for glioblastoma propagation (Suvà *et al*., 2014). Therefore, how SALL2 is associated with cancer is controversial. In addition, mechanisms and targets of SALL2 that could explain its role in disease are yet scarce (Hermosilla *et al*., 2017; Sung and Yim, 2017).

Here, we demonstrated that *Sall2* deficiency, in normal and immortal fibroblasts, triggers uncontrolled cell proliferation, which correlates with cell cycle alteration and increased tumorigenic potential of immortal cells *in vitro*. Our data are consistent with a role of SALL2 in cell cycle arrest and with previous studies in other cell types. Indeed, depletion of SALL2 (silencing) in human ovarian surface epithelial HOSE (*Sall2* expressing) cells increased DNA synthesis measured by BrdU incorporation (Li *et al*., 2004). On the other hand, gain of SALL2 function (overexpression) decreased DNA synthesis in SKOV3 (*Sall2*‐deficient) ovarian cancer cells (Li *et al*., 2004). SALL2 was also identified as a key factor for cellular quiescence of human foreskin fibroblasts, showing early upregulation of SALL2 upon serum deprivation (Liu *et al*., 2007). Silencing of SALL2 blocked the ability of cells to arrest in G0–G1 after growth factor deprivation, leading to inappropriate progression through S and G2‐M phases (Liu *et al*., 2007). In agreement, we found that asynchronous primary *Sall2* ^−/−^ MEFs present higher proportion of cells in S phase. However, we did not observe a notorious difference in the G2‐M phases between asynchronous (Fig. 1E 29.13% vs 21.72%) or nocodazole synchronized *Sall2* ^*+/+*^ and *Sall2* ^−/−^ MEFs (*t* = 0). Still, SALL2 levels are high in mitotic synchronized MEFs. These results suggest that SALL2 is also involved in regulation of mitotic exit, which could in part explain the accelerated progression of *Sall2* ^−/−^ cells into G1. As previous studies used siRNA pools and serum deprivation in human foreskin fibroblasts (Liu *et al*., 2007), or overexpression of SALL2 in—p16‐deficient—SKOV3 cells (Wu *et al*., 2015), the difference in the effect of SALL2 at G2‐M might relate to experimental conditions, cell‐type specificity or the genetic context. Whether the role of SALL2 in G2‐M is cell‐type dependent is out of the focus of the present study and should be analyzed in future studies. Nevertheless, nocodazole‐synchronized *Sall2* ^−/−^ iMEFs inappropriately progressed through the cell cycle, entering earlier than wild‐type iMEFs into G1 and S phases, which is associated with the increased proliferation of the *Sall2‐*deficient cells detected by FACs and BrdU incorporation studies, and consistent with the negative regulation of G1/S cyclins by SALL2.

Our current study shows for the first time that SALL2 represses cyclin D1 and cyclin E1 expression, further supporting a role of SALL2 during G1/S transition. Cyclins are sequentially expressed during the cell cycle, forming complexes with cyclin‐dependent kinases that phosphorylate target proteins required for progression through the cell cycle (Suryadinata *et al*., 2010). Cyclin types D and E play major roles at the G1‐ to S‐phase transition; specifically, the induction of cyclin D1 is a rate‐limiting event for cyclin‐dependent activation of CDK4 kinase and the subsequent transcriptional activation of cyclin E gene for progression beyond G1/S transition (Kim and Diehl, 2009). Type E cyclins express during late G1 phase until the end of the S phase and are limiting for the passage of cells through the restriction point ‘R’ from a resting state, or passing from G1 to S phase (Möröy and Geisen, 2004; Siu *et al*., 2012). Because of the relevance of cyclin D1 and cyclin E1 expression during cell cycle progression, both cyclins are highly regulated at specific times through transcriptional, post‐transcriptional, and post‐translational mechanisms (Kim and Diehl, 2009; Klein and Assoian, 2008; Möröy and Geisen, 2004; Qie and Diehl, 2016; Siu *et al*., 2012). Consistent with transcriptional repression, ectopic SALL2 (E1 or E1A isoform) repressed cyclin D1 and E1 promoter's activity. In addition, chromatin immunoprecipitation experiments showed specific binding of each SALL2 isoform to the proximal region of *CCND1* and *CCNE1* genes’ promoters. Of note, even though most of our studies were carried out in MEFs, which express mainly E1A isoform, ectopic expression of either E1 or E1A represses the transcriptional activities of cyclin promoters, suggesting that *CCND1* and *CCNE1* are not isoform‐specific but rather common targets.

**Figure 7:**
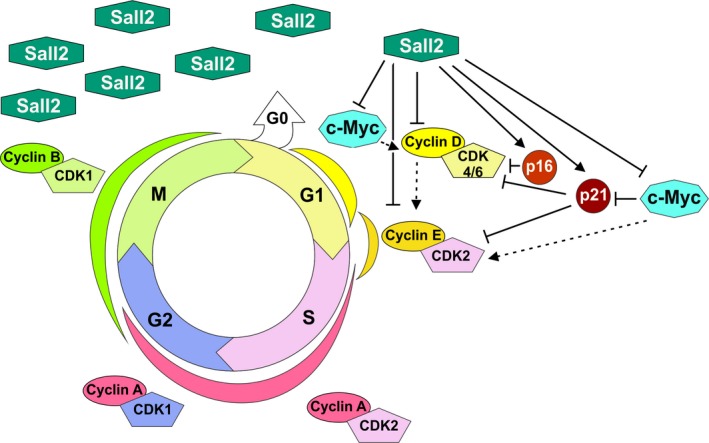
Model of SALL2‐dependent regulation of the cell cycle. SALL2 directly inhibits G1‐S phase progression by repressing cyclin D1 and cyclin E1 expression (this article). Additionally, SALL2 induces p21^CIF^ ^/^ ^WAF^ (p21) and p16^INK^ ^4a^ (p16) and represses c‐Myc. As c‐Myc represses p21, SALL2‐mediated c‐Myc repression could indirectly increase p21 levels. As Myc could trigger cyclin E1 and D1 expression, SALL2 could indirectly downregulate cyclins by repressing c‐Myc. Dotted arrow indicates indirect effects.

SALL2 isoforms differ in the first exon but share exon 2, which contains most of the protein sequence including the DNA binding and transactivation domains (Hermosilla *et al*., 2017). The repressor function of E1 could be explained by a conserved repressor domain present at its N‐terminal region, which binds to the NuRD complex (Lauberth and Rauchman, 2006). E1A does not contain this domain neither interacts with the NuRD complex (Lauberth and Rauchman, 2006); however, it has been reported that similar to the effect of SALL2 E1, the expression of SALL2 E1A represses several gene promoters activity, including *TK*,* hTERT*,* c‐Myc,* and *Sp1*. (Wu *et al*., 2015). How SALL2 E1A represses the cyclins promoter is at the present unknown, but might result from direct repression of promoter's activity. Similar to repression mediated by p53, E1A could bind to an element that overlaps—or are similar—to sites of activator/coactivator molecules, directly bind to promoter elements and disrupt the pre‐initiation complex assembly, or assemble a multiprotein repressor complex (van Bodegom *et al*., 2010; Bohlig and Rother, 2011; Ho and Benchimol, 2003; Zaky *et al*., 2008). Our ChIP studies suggest a direct repression of cyclin D1/E1 by SALL2, although they did not rule out an indirect effect through participation of other transcription factors or cofactors in the process. For instance, SALL2 transcriptionally represses c‐Myc (Sung *et al*., 2012). As c‐Myc regulates *CCND1* and *CCNE1* expression and p21^CIF/WAF^ (Jansen‐Dürr *et al*., 1993; Obaya *et al*., 1999; Coller *et al*., 2000; Gartel *et al*., 2001), it could be indirectly responsible for some of the effects of SALL2. However, our results argue against this possibility because we were unable to find SALL2‐dependent changes of c‐Myc expression in HEK293 cells (Fig. S4). On the other hand, cyclin D–Cdk4/6 complexes could lead to partial activation of E2F transcription factor, allowing the transcription of *CCNE1* gene (Suryadinata *et al*., 2010), suggesting that SALL2‐dependent cyclin D repression could lead to repression of cyclin E (Fig. 7). Although this pathway is clearly not involved in HEK293 cells where we failed to detect any change on cyclin E1 expression, it could contribute to SALL2‐dependent repression of cyclin E1 in MEFs. Nevertheless, our transcriptional studies strongly suggest that SALL2 directly regulates cyclin E1 expression.

Identification of other SALL2 transcriptional targets related to the control of cell proliferation comes mainly from overexpression experiments, which identified that SALL2 induces the expression of two cell cycle inhibitory proteins: cyclin‐dependent kinase inhibitor I (*CDKN1A*, p21^CIF/WAF^) (Li *et al*., 2004) and cyclin‐dependent kinase 4 inhibitor A (*CDKN2A*, p16^INK4a^) (Wu *et al*., 2015) (Fig. 7). As p16^INK4a^ blocks cyclin D‐Cdk4/6 complexes during G1 phase (Serrano *et al*., 1993), and p21 blocks cyclin E/Cdk2 complex at the G1/S check point (Harada and Ogden, 2000; Harper *et al*., 1995), SALL2 regulation of previously reported targets also supports its role during G1‐/S‐phase transition.

We showed that *Sall2* deficiency associates with increased expression of Cyclin D1 and E1 (at mRNA and protein levels), but does not affect the expression and/or kinetics of cyclin A and B1 through the cell cycle progression. Therefore, the deregulation of cyclins D1 and E1 is likely involved in the proliferative advantage of the *Sall2* ^−/−^ MEFs. Relevant is that the inverse correlation between SALL2 and cyclin D1/E1 expression in MEFs is also confirmed in tissues from wild‐type and *Sall2* knockout mice, suggesting that SALL2 repression of cyclin D1 and E1 expression occurs *in vivo*. These results open the possibility of SALL2 association with other functions of cyclins. In particular, in addition to its role as a CDK‐dependent regulator of the cell cycle, cyclin D1 has also CDK‐independent functions associated with the regulation of cellular metabolism, cell differentiation, and cellular migration (Dai *et al*., 2013; Fu *et al*., 2005; Pestell, 2013). It is particularly intriguing that SALL2 and cyclin D1 have both been associated with neurogenesis (Bohm *et al*., 2007; Pincheira and Donner, 2008; Pogoriler *et al*., 2006).

Considering only the transcriptional aspect of the regulation of cyclin D1 expression, our results suggest that SALL2 controls the steady‐state levels of cyclin D1, but changes in the activity of other transcriptional repressors and/or activators surely contributes in the increase in cyclin D1 (Klein and Assoian, 2008). Several positive and negative regulators of cyclin D1 expression are known, and these include transcription factors that directly bind and repress or activate *CCND1* promoter, such as ATF3, a member of the AP1 family that represses cyclin D1 expression (Lu *et al*., 2006). Besides ATF3, other factor such as SMAR1 inhibits cyclin D1 transcription by recruiting to the *CCND1* promoter a repressor complex containing Sin3, HDAC1, and the pocket proteins p107 and p130 (Rampalli *et al*., 2005). Other identified *CCND1* repressors include ZO‐2 and p19^ARF^ (D'Amico *et al*., 2004; Huerta *et al*., 2007). In relation to cyclin E1 transcriptional repression less information is available. Repression of *CCNE1* gene during G2‐M and the early G1 phases of the cell cycle is mediated through the assembly of a multiprotein complex containing hypophosphorylated pRb, HDAC, and SWI/SNF, which is recruited to E2F transcription factors to the *CCNE1* promoter to silence transcription (Möröy and Geisen, 2004; Suryadinata *et al*., 2010; Zhang *et al*., 2000). In addition, studies identified Wilms tumor 1 (WT1) (Loeb *et al*., 2002), NF‐kB (p65/RelA) (Janbandhu *et al*., 2010), and NFAT (Teixeira *et al*., 2016) transcription factors as negative regulators of *CCNE1* gene expression. How all these factors are coordinated with the regulation of cyclins by SALL2 as well as the underlying mechanism of SALL2 transcriptional repression will require further investigation.

Interesting for cancer research, it is the fact that *Sall2* deficiency increases the growth rate, foci formation, and ability of immortalized MEFs to form colonies in soft agar, similar to the effect of knocking out *ATF3*, an inhibitor of cyclin D1 (Lu *et al*., 2006). In addition, we found a significant inverse correlation between *CCND1/E1* and *SALL2* expression in various cancers, but most notoriously between *CCNE1* and *SALL2* in breast cancer. Analysis of four independent breast cancer datasets showed that *SALL2* mRNA inversely correlates with *CCNE1* mRNA levels. Although further studies are needed to address whether these correlations are consequence of a functional deficiency of SALL2, it is intriguing that upregulation of cyclin E1 has been reported in many cancer types, and most carefully investigated in breast cancer (Barton *et al*., 2006; Hwang and Clurman, 2005; Inoue and Fry, 2016; Keyomarsi *et al*., 2002; Möröy and Geisen, 2004). The mechanisms associated with cyclin E1 overexpression include deregulation of RB pathway by mutations on its regulators, which increased E2F activity, *CCNE1* gene amplification, and disrupted proteolysis (Siu *et al*., 2012). Cyclin E1 (full length and short form) over expression correlates with poor clinical outcome and greatest risk of recurrence (Hunt *et al*., 2017; Keyomarsi *et al*., 2002). In relation to SALL2 and breast cancer, two independent bioinformatic studies identified SALL2 as a highly relevant breast cancer biomarker (Liu *et al*., 2014; Zuo *et al*., 2017). The studies suggest SALL2 as a putative shared target gene of MYB and ARNT2 transcription factors and as putative suppressor of epithelial–mesenchymal transition activities. SALL2 is identified as one of the candidate drivers for attenuating histological grade promotion, and in preventing cancer progression (Liu *et al*., 2014; Zuo *et al*., 2017). Our findings suggest a novel mechanism through *CCND1/E1* promoter derepression by loss/deficiency of SALL2 tumor suppressor function. However, it is intriguing the inverse correlation between *SALL2/CCNE1* in glioblastoma, as SALL2 was identified as a factor that promotes glioblastoma propagation (Suvà *et al*., 2014). In addition, the correlation between SALL2 and *CCND1* expression was ambiguous, finding positive and negative correlations depending on the cancer type. This suggests that other factors, genetic context or tissue specificity, are involved in the regulation of *CCND1/E1* by SALL2. This possibility is consistent with Fig. 4A,C and D that shows inverse correlation only in a subset of normal mouse tissues. Additional clinical and basic studies are needed to support the role of SALL2 as a tumor suppressor in breast cancer, lymphoma, cervix, pancreas, colon, and/or lung cancer.

In summary, we presented evidence of a role of SALL2 in the inhibition of the cell cycle during G1‐ and G1‐ to S‐phase transition. Together with the results showing increased tumorigenic potential of *Sall2* ^−/−^ cells, our studies indicate a potential mechanistic association of *SALL2* deficiency with cancer, through loss of a tumor suppressor function. In further support of this function, there are the previous studies demonstrating that SALL2 transcriptionally increases p21 and p16 Cdk inhibitors’ expression and that SALL2 represses the proto‐oncogene c‐Myc. We also previously demonstrated that SALL2 induces apoptosis of MEFs and human leukemia cells exposed to genotoxic stress (Escobar *et al*., 2015). Still, SALL2 upregulation in some types of cancer seems to be inconsistent with a tumor suppressor role (Alagaratnam *et al*., 2011; Estilo *et al*., 2009; Li *et al*., 2002; Nielsen *et al*., 2003; Suvà *et al*., 2014). However, the *SALL2* gene status, subcellular localization, and/or SALL2 isoform expression in those cancers are unknown, which could shed light on the apparently conflicting results. Also, we cannot rule out other SALL2 functions associated with other cellular contexts, targets, and/or cell types. In this sense, there is evidence indicating that SALL2 is involved in stemness (Hermosilla *et al*., 2017), a function that may explain its role in reprogramming differentiated glioblastoma cells into those with ability to propagate a tumor *in vivo*. Nevertheless, the revealed function of SALL2 on the repression of cyclins D1 and E1 opens a new perspective for understanding not only SALL2 association with disease, but also its normal function.

## 6 Conclusions

SALL2 inhibits cell proliferation by repressing G1‐ to S‐phase cell cycle transition. The effect of SALL2 on cell cycle progression is associated with the transcriptional repression of cyclins D1 and E1 expression. Accordingly, SALL2 behaves as a tumor suppressor.

## Author contributions

RP conceived and coordinated the project. VEH, GS, ER, DE, MIH, and CF performed experiments and acquisition of data. VEH and RP performed overall data interpretation. MG, VM, MAG, and AFC helped with data interpretation and critically reviewed the manuscript, and VEH, MG, AFC, and RP wrote the manuscript. All authors read and approved the final version of the manuscript.

## Supporting information


**Fig. S1** *Sall2* deficiency does not contribute to immortalization of MEFs.

**Fig. S2.** Characterization of iMEFs.

**Fig S3.** Increased BrdU incorporation of *Sall2*‐deficient iMEFs.

**Fig S4.** Loss/gain of SALL2 function inversely correlated with levels of cyclin D1 in HEK293 cells.

**Fig. S5.** DNA sequences of human and mouse proximal promoter regions of *CCND1* and *CCNE1*.

**Table S1.** Inverse correlation between *SALL2* and *CCNE1/D1* expression in various cancers.
