# Supplementary figures and images for: SALL2 represses cyclins D1 and E1 expression and restrains G1/S cell cycle transition and cancer‐related phenotypes
Source: Mol Oncol. 2018 May 21;12(7):1026–46. doi: 10.1002/1878-0261.12308 (PMC6026872; doi:10.1002/1878-0261.12308)

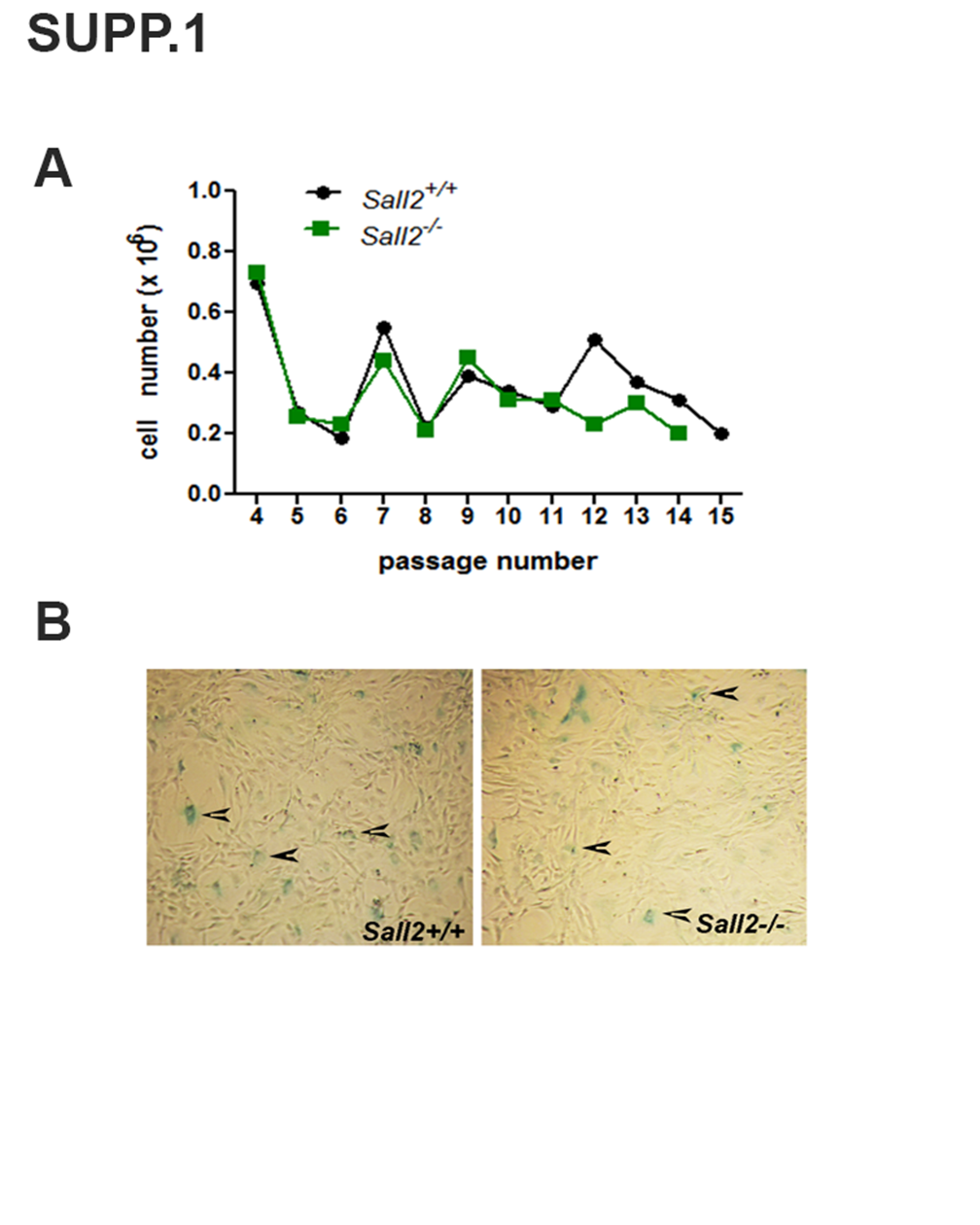

Supplement: Supplementary file 1 — Fig. S1 Sall2 deficiency does not contribute to immortalization of MEFs. [file MOL2-12-1026-s001.tif]

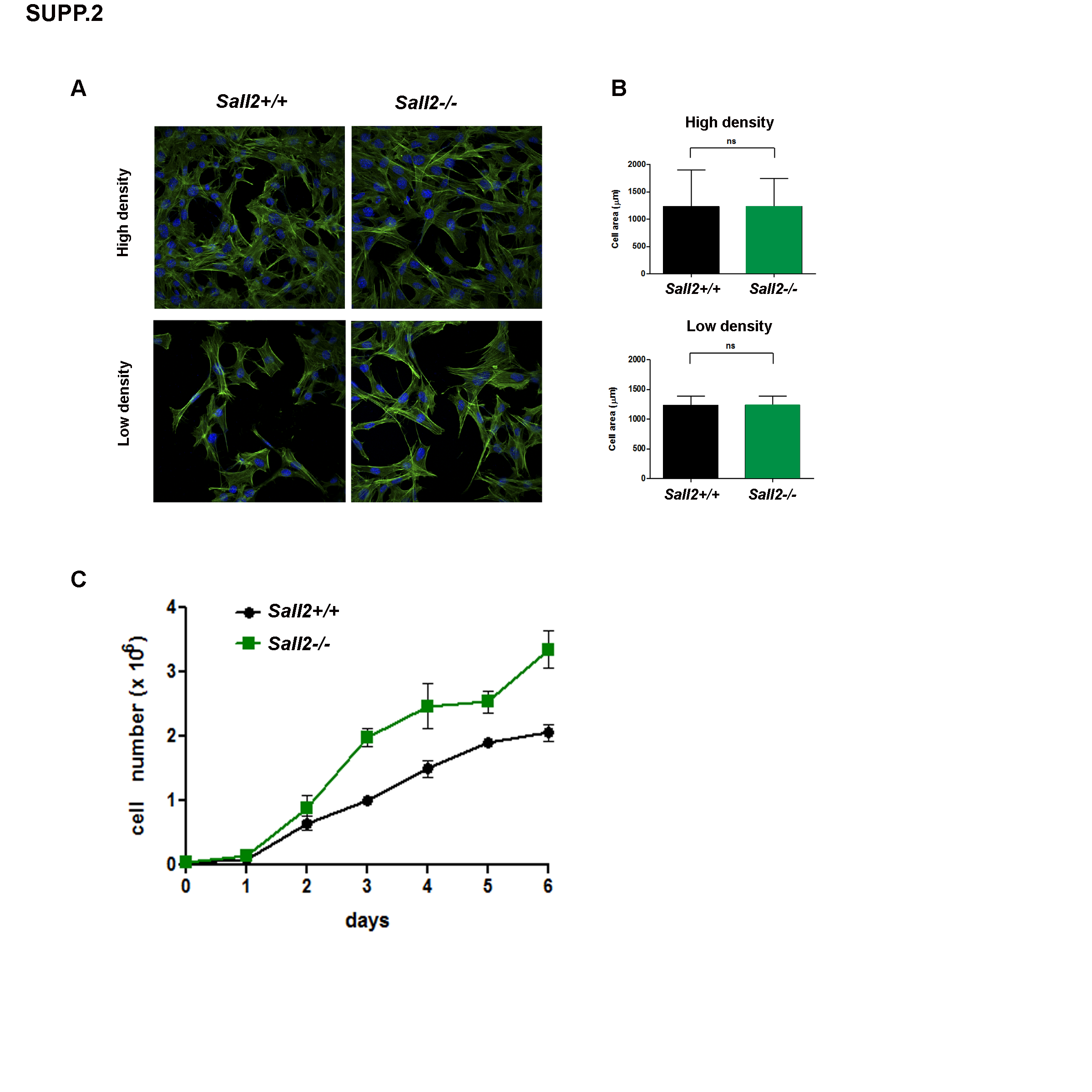

Supplement: Supplementary file 2 — Fig. S2. Characterization of iMEFs. [file MOL2-12-1026-s002.tif]

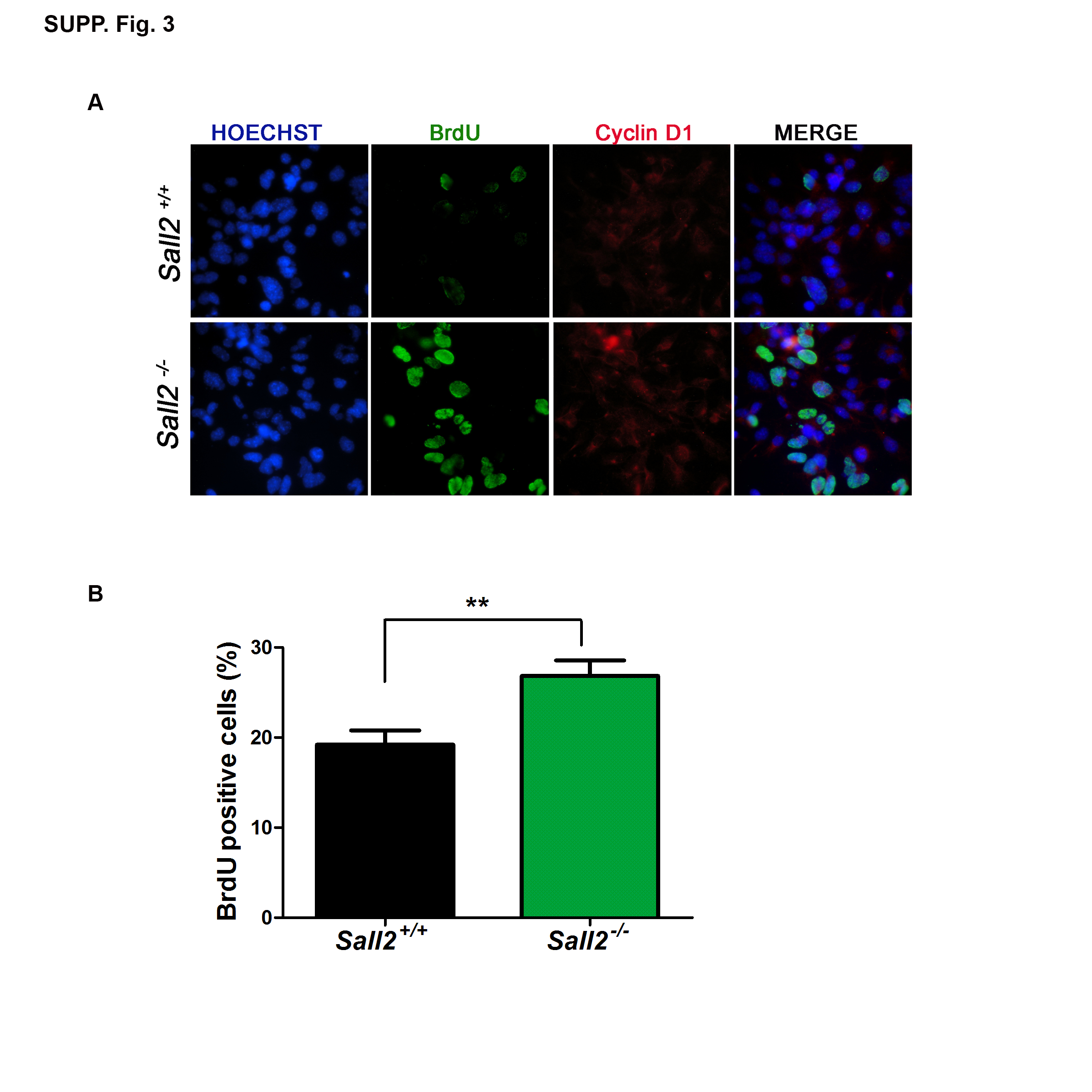

Supplement: Supplementary file 3 — Fig S3. Increased BrdU incorporation of Sall2‐deficient iMEFs. [file MOL2-12-1026-s003.tif]

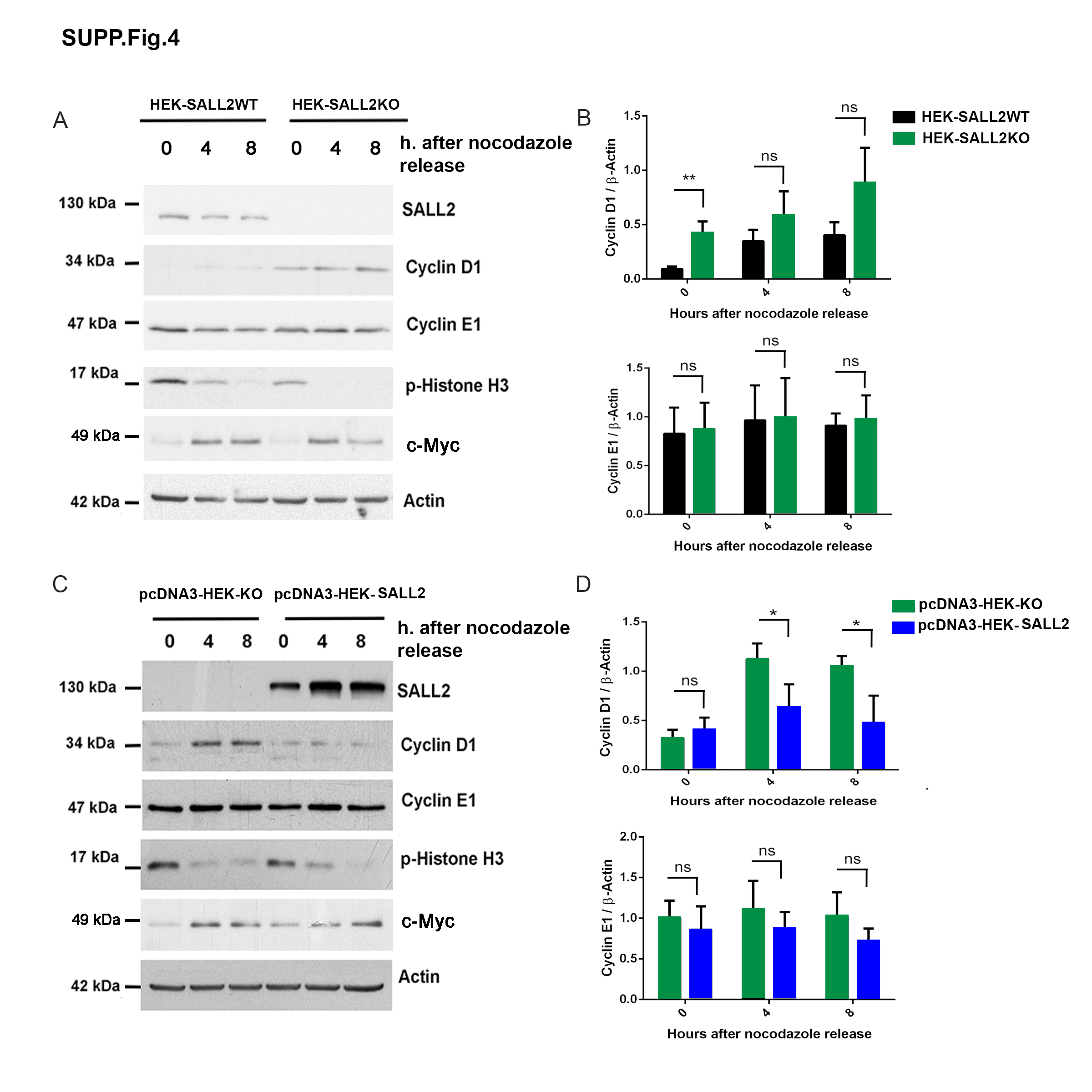

Supplement: Supplementary file 4 — Fig S4. Loss/gain of SALL2 function inversely correlated with levels of cyclin D1 in HEK293 cells. [file MOL2-12-1026-s004.tif]

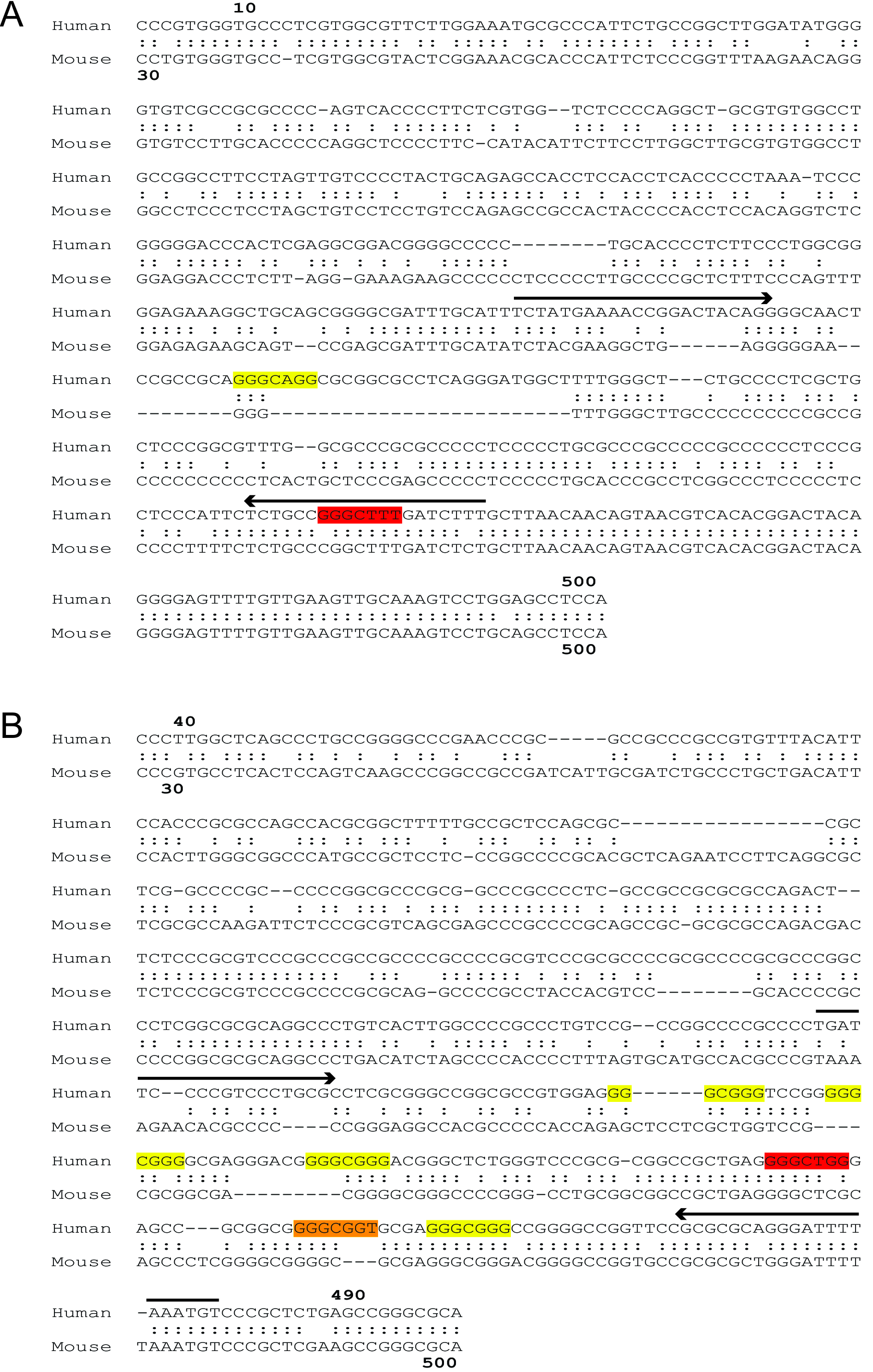

Supplement: Supplementary file 5 — Fig. S5. DNA sequences of human and mouse proximal promoter regions of CCND1 and CCNE1. [file MOL2-12-1026-s005.tif]
