# Supplementary material for: SALL2 represses cyclins D1 and E1 expression and restrains G1/S cell cycle transition and cancer‐related phenotypes
Source: Mol Oncol. 2018 May 21;12(7):1026–46. doi: 10.1002/1878-0261.12308 (PMC6026872; doi:10.1002/1878-0261.12308)
Supplement: Supplementary file 6 — Table S1. Inverse correlation between SALL2 and CCNE1/D1 expression in various cancers. [file MOL2-12-1026-s006.pdf]

	SALL2/***CCNE1***			*SALL2*/***CCND1***			
**Cancer type**	**r**	**p Value**	**n**	**R**	**p Value**	**n**	**GEO ID**
**Cervix Cancer**	-0.440	4.0e-03	41	0.390	0.01	45	GSE7803
**Cervix Cancer**	-0.393	1.1e-03	66	0.150	0.23	66	GSE9750
**Endometrial Cancer**	-0.560	1.8e-07	75	-0.510	3.0e-06	75	GSE11869
**Glioblastoma**	-0.410	2.0e-05	101	-0.611	1.2e-11	101	GSE4536
**Glioblastoma**	-0.316	7.6e-03	70	0.077	0.53	70	GSE34271
**Leukemia**	-0.373	4.6e-03	56	0.316	0.02	56	GSE4228
**Leukemia**	-0.026	0.24	2004	-0.110	7.5e-07	2004	GSE13159
**Leukemia**	-0.056	0.20	525	0.157	3.2e-04	525	GSE14468
**Lymphoma**	-0.213	1.7e-03	215	-0.040	0.56	215	GSE4475
**Lymphoma**	-0.203	5.5e-04	287	0.043	0.46	287	GSE23120
**Lymphoma**	-0.579	3.3e-04	34	0.372	0.03	34	GSE34771
**Osteosarcoma**	0.507	0.02	20	-0.049	0.84	20	GSE14359
**Bladder Cancer**	-0.016	0.90	60	-0.332	9.6e-03	60	GSE3167
**Ovarian Cancer**	-0.081	0.06	527	0.074	0.09	527	GSE68661
**Ovarian Cancer**	-0.313	0.04	45	-0.310	0.04	45	GSE38666
**Pancreatic Cancer**	-0.441	1.9e-03	47	-0.550	6.2e-05	47	GSE17891
**Breast Cancer**	-0.494	8.0e-17	251	0.259	3.3e-05	251	GSE3494
**Breast Cancer**	-0.322	6.3e-10	351	0.261	7.5e-07	351	GSE2109
**Breast Cancer**	-0.407	4.9e-12	262	0.417	1.3e-12	262	GSE21653
**Breast Cancer**	-0.349	1.3e-09	286	0.382	2.4e-11	286	GSE2034
**Colon Cancer**	-0.275	3.4e-08	390	-0.227	6.2e-06	390	GSE41258
**Colon Cancer**	-0.267	1.5e-06	315	0.054	0.34	315	GSE2109
**Colon Cancer**	-0.299	5.5e-04	130	-0.218	0.01	130	GSE37892
**Kidney Cancer**	-0.198	1.3e-03	261	0.031	0.62	261	GSE2109
**Liver Cancer**	0.262	0.01	90	0.159	0.13	90	GSE15765
**Lung Cancer**	-0.439	7.5e-03	36	-0.121	0.48	36	GSE14018
**Lung Cancer**	-0.280	4.8e-03	100	-0.014	0.89	100	GSE33532
**Lung Cancer**	-0.255	1.6e-03	159	0.037	0.65	159	GSE43580
**Sarcoma**	0.220	0.02	117	0.727	1.7e-20	117	GSE17679
**Thyroid Cancer**	-0.340	0.05	34	0.239	0.17	34	GSE2109
**Wilm's**	-0.162	0.05	144	-0.150	0.07	144	GSE10320

Supplementary Table 1. Inverse correlation between *SALL2* and *CCNE1/D1* expression in various cancers
